# Practice beats age: co-activation shapes heritage speakers’ lexical access more than age of onset

**DOI:** 10.3389/fpsyg.2023.1141174

**Published:** 2023-06-12

**Authors:** Nuria Sagarra, Joseph V. Casillas

**Affiliations:** Department of Spanish and Portuguese, Rutgers University, New Brunswick, NJ, United States

**Keywords:** heritage speakers, stress, age of onset, proficiency, use, eye-tracking, lexical access, co-activation

## Abstract

Probabilistic associations make language processing efficient and are honed through experience. However, it is unclear what language experience factors explain the non-monolingual processing behaviors typical of L2 learners and heritage speakers (HSs). We investigated whether AoO, language proficiency, and language use affect the recognition of Spanish stress-tense suffix associations involving a stressed syllable that cues a present suffix (*SALta “*s/he jumps”) and an unstressed syllable that cues a past suffix (*SALtó “*s/he jumped”). Adult Spanish-English HSs, English-Spanish L2 learners, and Spanish monolinguals saw a paroxytone verb (stressed initial syllable) and an oxytone verb (unstressed initial syllable), listened to a sentence containing one of the verbs, and chose the one they heard. Spanish proficiency measured grammatical and lexical knowledge, and Spanish use assessed percentage of current usage. Both bilingual groups were comparable in Spanish proficiency and use. Eye-tracking data showed that all groups fixated on target verbs above chance before hearing the syllable containing the suffix, except the HSs in the oxytones. Monolinguals fixated on targets more and earlier, although at a slower rate, than HSs and L2 learners; in turn, HSs fixated on targets more and earlier than L2 learners, except in oxytones. Higher proficiency increased target fixations in HSs (oxytones) and L2 learners (paroxytones), but greater use only increased target fixations in HSs (oxytones). Taken together, our data show that HSs’ lexical access depends more on number of lexical competitors (co-activation of two L1 lexica) and type (phonotactic) frequency than token (lexical) frequency or AoO. We discuss the contribution of these findings to models in phonology, lexical access, language processing, language prediction, and human cognition.

## 1. Introduction

Monolinguals use multiple cues to predict what a speaker will say, but L2 learners struggle when making predictions based on L2 cues absent in their L1. However, it is unclear what causes this struggle. The study of heritage speakers (HSs) allows us to examine the role of age of onset (AoO) and language experience on L2 processing. These variables advance our understanding of why HSs differ from monolinguals and first-generation immigrants. HSs refer to “individuals from minority language groups who grow up exposed to a minority language in the home and the majority societal language” (Montrul, 2016, p. 16). HSs’ uniqueness is attributed to representational differences (Montrul, 2008), limited quality input (Pires and Rothman, 2009), gradual attrition (Polinsky, 2011), or reduced current activation of their heritage language (Putnam and Sánchez, 2013). We explored alternative explanations in terms of reduced knowledge of their heritage language (proficiency), as well as increased lexical competition due to co-activation of their two L1 lexica (use: current amount of input, output, and interaction in a language on a weekly basis). We employed an auditory implicit eye-tracking task and investigated whether AoO, language proficiency, and language use influence how monolinguals, HSs, and L2 learners form stress-suffix lexical associations during spoken word access. Probabilistic associations are crucial in making spoken language processing efficient (Romberg and Saffran, 2010), and are refined through experience. L2 studies show that higher language proficiency facilitates tone-tense and tone-number suffix associations in L2 Swedish (Schremm et al., 2016; Gosselke Berthelsen et al., 2018, 2020) and stress-tense suffix associations in L2 Spanish (Sagarra and Casillas, 2018), and that novice learners only recognize L2 tone-suffix associations if their L1 is tonal (Gosselke Berthelsen et al., 2021).

While research comparing monolinguals to both L2 learners and HSs could tease apart AoO from language experience, these studies are often inconclusive. Written mode studies (e.g., Foote, 2011; Keating, 2022; Parshina et al., 2022) are problematic because HSs perform auditory tasks better than written ones (Bowles, 2011). Single-proficiency studies are unable to determine whether non-native processing is due to late AoO, low proficiency, or both (e.g., Sekerina and Trueswell, 2011; Montrul et al., 2013; Jegerski and Sekerina, 2020). L2 studies without HSs (e.g., Nichols and Joanisse, 2016), HS studies without monolinguals (Lemmerth and Hopp, 2019), or HS studies with a composite score merging AoO and proficiency (Hervais-Adelman et al., 2018) are incapable of assessing AoO effects. Some studies combined AoO and proficiency (Wartenburger et al., 2003; Hervais-Adelman et al., 2018; Sagarra and Rodríguez, 2022), AoO and use (Lloyd-Smith et al., 2019), or proficiency and use (Di Pisa and Marinis, 2022), and the only study examining AoO, proficiency and use separately examined morphosyntax (Sagarra et al., 2021). We investigated the separate effects of AoO, proficiency, and current use on the recognition of Spanish stress-tense suffix associations by Spanish-English HSs, English-Spanish L2 learners, and Spanish monolinguals. Lexical stress is contrastive in English and Spanish, but these languages differ in stress realization and cue weight.

## 2. Lexical stress

Lexical stress (henceforth stress) refers to the relative prominence of one syllable with regard to the others in a given word. Stress is lexically encoded and contrastive in Spanish (*término* [ˈteɾ.mi.no] “term;” *termino* [teɾ.ˈmi.no] “I finish;” *terminó* [teɾ.mi.ˈno] “s/he finished”) and in English (*produce* [ˈpɹo.duːs] noun; *produce* [pɹə.ˈduːs] verb), though it is more productive in Spanish than in English. To wit, few stress minimal pairs exist in English that are not semantically related (see Cutler, 2012). The primary acoustic correlates of stress are f0, duration, and intensity, although their relative cue-weighting is language-specific (see Holt and Lotto, 2006; Chrabaszcz et al., 2014; Gordon and Roettger, 2017, among many others). Despite native English speakers’ familiarity with stress, they typically have trouble producing (Bullock and Lord, 2003; Lord, 2007) and perceiving (Face, 2000, 2005, 2006; Saalfeld, 2012; Ortega-Llebaria et al., 2013) stress differences in L2 Spanish. A possible explanation might be found in language-specific isochrony (Pike, 1945). Whereas English is often described as a “stress-timed” language, i.e., one with relatively constant intervals between stressed syllables, Spanish is typically described as “syllable-timed,” i.e., each syllable is perceived as having the same duration. Differences such as these may shape how stress is perceived in each language. In English, for example, unstressed vowel reduction—often present in stress-timed languages—may be sufficient for indicating stress (Cutler, 2012; Tremblay et al., 2018), rendering other cues relatively less important for speech perception. Consequently, native English speakers need to adjust their cue-weighting strategies when learning Spanish, a language that does not have vowel reduction. Evidence from cross-modal priming studies indicates that stress is processed differently by native listeners in both languages during lexical access (see Soto-Faraco et al., 2001; Cooper et al., 2002). Extant literature also suggests that native listeners are tuned in to the relevant acoustic cues of their language and take advantage of them to increase processing efficiency. Unsurprisingly, they use the same cue-weighting strategies when learning an L2, which often generates difficulties in the early stages of acquisition (Iverson et al., 2003; Ingvalson et al., 2012). With respect to prediction, there is evidence that monolingual Spanish speakers use lexical stress to predict a word’s suffix and that highly proficient L2 learners can also master this skill (Sagarra and Casillas, 2018), but it is unclear whether unique language experiences and earlier AoO modulate spoken word prediction.

## 3. The role of AoO, proficiency, and use on bilingual language processing and prediction

Hundreds of studies conducted over half a century have yielded mixed findings regarding the effects of AoO on language acquisition in bilinguals (see Mayberry and Kluender, 2017, for a review, and Singleton and Leśniewska, 2021, for an argument that the critical period hypothesis is irrelevant because it is unfalsifiable). Offline studies are inconclusive. Some studies showed that advanced HSs were grammatically more accurate than advanced L2 learners in perception and production tasks (Bowles, 2011), whereas others did not reveal any grammar differences between the two at any proficiency level (Foote, 2011). Relevant to our study, Kim (2020) reported that Spanish-English HSs perceived Spanish lexical stress more accurately than English-Spanish L2 learners, but the two were equally deviant from monolinguals in production. Online studies are equally ambiguous. While several studies concluded that HSs processed morphology more effectively with earlier than later AoO (Veríssimo et al., 2018), others showed no differences between HSs and L2 learners (Wartenburger et al., 2003; Foote, 2011; Rodríguez and Reglero, 2015; Martohardjono et al., 2017). This lack of consensus has led researchers to question if we are missing the point by focusing on AoO rather than the quality and quantity of bilinguals’ individual language experiences (Luk and Pliatsikas, 2015). Although bilinguals and monolinguals are conceived as separate homogeneous groups, the degrees of variability among bilinguals are enormous (De Bruin, 2019). The investigation of language proficiency and use advances our understanding of what factors produce such variability.

L2 proficiency studies showed that low L2 proficiency denoted delayed processing, insensitivity to violations, processing violations as semantic anomalies, reduced attention to cues used by monolinguals, and less and later fixations on targets (see Ito and Pickering, 2021, for a review of L2 prediction studies examining proficiency effects). Regarding morphology, higher L2 proficiency promoted the processing of L2-derived and inflected words, new valid derivations, and forms combining a real stem with a new suffix (Kimppa et al., 2019). Concerning phonology, higher L2 proficiency inhibited L1 lexical activation (Berghoff et al., 2021), facilitated the distinction of L2 phonemic contrasts (White et al., 2015), and increased monolingual-like pronunciation (Maddah and Reiterer, 2018), intonation (Jun and Oh, 2000) and stress (Konishi et al., 2018). Furthermore, neural representations change with L2 proficiency (see Pliatsikas et al., 2020, for a review) and higher proficiency L2 learners activate the same areas in the brain as monolinguals (Vingerhoets et al., 2003). Though numerous studies investigated the role of proficiency with late bilinguals, to our knowledge, only five online studies examined proficiency in early bilinguals. Bice and Kroll (2021) investigated the role of proficiency and working memory on grammatical judgments in HSs and monolinguals. They found that HS showed smaller P600 and N400 effects (i.e., sensitivity to syntactic and semantic violations) than monolinguals, and that ERP variation for grammatical judgments was mostly caused by proficiency (a fluid variable) in the HSs and by working memory (a stable variable) in the monolinguals: Wartenburger et al. (2003), Hervais-Adelman et al. (2018), Sagarra and Rodríguez (2022), and Sagarra et al. (2021) also reported how proficiency affected the ways that HSs processed their heritage language. Because these studies investigated proficiency and AoO, we cover them at the end of the background section as part of our review of studies that investigate multiple language experience variables. Taken together, L2 and HS studies suggest that higher proficiency facilitates morphosyntactic and syntactic processing. Although many studies examined the role of proficiency during language processing in bilinguals, only a few studies explored usage-based measures (Surrain and Luk, 2019). Next, we will summarize bilingual studies including these measures.

Language use is an important component of language processing and acquisition (Ranta and Meckelborg, 2013). L2 studies showed that greater L2 use facilitated monolingual-like L2 morphosyntactic processing (Faretta-Stutenberg and Morgan-Short, 2018), sensitivity to gender code-switching rules (Beatty-Martínez et al., 2020), L2 grammar development (Isabelli-García and Lacorte, 2016), L2 auditory production (Muñoz, 2014), reduction of foreign accents (Abu-Rabia and Kehat, 2004), and discrimination of consonants (Black et al., 2020) and vowels (Flege and MacKay, 2004). Similarly, HS studies demonstrated that greater language use facilitated monolingual-like syntax (Schmidd, 2022), pronunciation (Lloyd-Smith et al., 2019) and reduction of foreign accents (Yeni-Komshian et al., 2000). Pereira Soares (2022) reported that early AoO and greater language use increased functional brain connectivity in HSs and L2 learners; however, the HSs showed greater connectivity and inhibitory control than the learners. Four online HS studies did not measure language use, but their findings appeared to be attributable to language use and exposure. These HS studies employed written tasks, using self-paced reading (Foote, 2011), eye-tracking (Keating, 2022; Parshina et al., 2022), and ERPs (Caffarra et al., 2017). In Foote, HSs and bilingual native speakers raised abroad were equally sensitive to gender and number agreement violations. In Keating, sequential bilinguals were more perceptive to gender agreement violations than simultaneous bilinguals because sequential bilinguals typically use their heritage language longer than simultaneous bilinguals. In Parshina et al., HSs and L2 learners predicted the gender of an upcoming noun, while only the HSs predicted its number; importantly, the HSs benefited from higher literacy experience. Finally, Caffarra et al. found that gender to gender agreement violations increased with greater language use for opaque nouns (opaque nouns mark gender lexically), but with higher language dominance mostly for transparent nouns (transparent nouns mark gender morphologically). Because HSs perform worse in written than auditory tasks (Bowles, 2011), it is important to examine the four HS studies employing auditory eye-tracking tasks to investigate syntactic predictions (Sekerina and Trueswell, 2011; Jegerski and Sekerina, 2020) and morphosyntactic predictions (Fuchs, 2021; Sagarra et al., 2021). In Sekerina and Trueswell, HSs were slower in processing contrastive focus than monolinguals, due to the HSs having used their heritage language less than the monolinguals. In Jegerski and Sekerina, HSs and L2 learners raised abroad were equally sensitive to the Spanish object marker *a*, showing that using Spanish for a longer period of time can compensate for a later AoO. In Fuchs (2021, 2022), HSs and native speakers of Spanish and Polish used lexical gender cues to make gender agreement predictions. Considering that native speakers use lexical gender cues even with gender transparent nouns (Zeller et al., 2022) and that L2 learners struggle using these cues (see Lemmerth and Hopp, 2019, for a review), we can conclude that the HS advantage over the L2 learners must be due to the HSs’ more extensive experience with their heritage language. However, Fuchs did not measure proficiency or use and could not determine whether their HS advantage was due to an earlier AoO, higher proficiency (proficiency was measured with self-reports and with accuracy in producing nouns with the correct gender), or greater frequency of use. Sagarra and Varela addressed this limitation by teasing apart the effects of AoO, proficiency and frequency of use. We describe this study at the end of the background section.

The studies reviewed thus far investigated the role of AoO, proficiency, or use, on bilingual language processing and learning in separate sample pools. Studies that have examined these variables within the same pool have produced different outcomes. We first review studies with L2 learners. Muñoz (2014) found that higher L2 use promoted L2 auditory production more than AoO. Hartshorne et al. (2018) reported that the effects of age, years of experience, and age of exposure in 680,333 participants revealed a late critical period of 17.4 years old to acquire new syntax. In contrast with these two offline studies, L2 neurocognitive studies offer a consistent picture regarding the benefits of greater language use. For instance, white matter microstructure—linked to improved nerve-impulse conduction and working memory function—changed: (a) with greater L2 use, rather than with earlier AoO or higher L2 proficiency (Del Maschio et al., 2020); and (b) with *later* AoO, a clear consequence of L2 use (Nichols and Joanisse, 2016; DeLuca et al., 2019). Similarly, subcortical structures associated with language control are shaped by longer L2 use (DeLuca et al., 2019). Relevant to our study, Fedeli et al. (2021) observed different effects of AoO, proficiency, and use on structural adaptations in the brain: AoO and L2 use modulated brain areas related to cognitive control, L2 proficiency affected those linked to word learning and language selection, and L2 use influenced those involved in overall comprehension and production. Taken together, the L2 studies reviewed in this paragraph suggest that offline techniques are not sensitive to all language experience nuances and that AoO, proficiency and use should be investigated separately within the same sample pool, because they restructure the brain differently. Next, we review studies combining AoO and proficiency (Wartenburger et al., 2003; Hervais-Adelman et al., 2018; Sagarra and Rodríguez, 2022), AoO and use (Lloyd-Smith et al., 2019), proficiency and use (Di Pisa and Marinis, 2022), and AoO, proficiency, and use (Sagarra et al., 2021).

Klein et al. (2014) found that bilinguals from birth had a similar brain structure to monolinguals: bilinguals with onset of 3–4 years and later showed thicker cortex in Broca’s area. In the same line, Hervais-Adelman et al. (2018) investigated AoO and proficiency effects in bilinguals speaking three or more languages. Greater “multilingual experience”—a composite variable formed by adding AoO (earlier receiving higher weight) and proficiency (more receiving higher weight)—enlarged brain structures associated with language control processes. Because AoO and proficiency were merged, participants with earlier AoO and greater proficiency were treated the same as those with later AoO and less proficiency. Wartenburger et al. (2003) addressed this limitation when examining the effects of proficiency and AoO on grammatical and semantic judgments in HSs and L2 learners with different proficiency levels. Proficiency and AoO affected the neural substrates of L2 processing, but proficiency shaped semantics whereas AoO modulated grammar. These results applied to an explicit task (judgments). Sagarra and Rodríguez (2022) explored the role of AoO and proficiency using an implicit reading eye-tracking task to assess how monolinguals, HSs, and L2 learners processed adjacent subject-verb number agreement. Monolinguals and HSs used articles to a greater extent than L2 learners regardless of proficiency, monolinguals and L2 learners fixated longer on more salient plural and preterit suffixes than less salient singular and present suffixes, and HSs were immune to plural-singular differences. This study did not measure language use, and the written task may have been too challenging for the HSs, because HSs perform poorly on written tasks. For example, HSs are more sensitive to grammatical violations than L2 learners in reading and speaking tasks, but HSs perform worse than L2 learners when completing writing tasks (e.g., Montrul et al., 2013, 2014).

Lloyd-Smith et al. (2019), Di Pisa and Marinis (2022), and Sagarra et al. (2021) addressed these limitations by measuring language use and by employing an auditory task. In Lloyd-Smith et al., Italian monolinguals, German-Italian L2 learners, and Italian-German HSs completed accent rating tasks in Italian and German. All groups were similar in German, but HSs’ perceived accent in Italian laid between the monolinguals and the learners. Majority language use did not affect HSs’ majority language or heritage language, and heritage language use did not affect HSs’ majority language; however, greater heritage language use clearly increased monolingual-like perception of heritage language accent. In Di Pisa and Marinis, Italian controls and HSs completed an elicited production task and a gender assignment task. Higher proficiency increased monolingual-like gender assignment and agreement, but higher use of the heritage language in the home only facilitated gender assignment. In Sagarra and Varela, Spanish monolinguals, and HSs and L2 learners of Spanish listened to sentences with determiner-noun-adjective gender agreement/disagreement while looking at a masculine and a feminine adjective on the screen. The two bilingual groups differed in AoO (before or after puberty) but were matched in proficiency (based on a Spanish proficiency test) and use (weekly percentage of Spanish input, output and interaction). Eye-tracking data revealed that monolinguals predicted earlier than bilinguals and HSs earlier than L2 learners, and that only the L2 learners struggled using lexical cues (knowing the gender of opaque-gender nouns) and attending to redundant syntactic cues (i.e., suffixes). While higher proficiency and use—but not earlier AoO—produced more predictions in both bilingual groups, these factors affected predictions differently: higher proficiency produced faster predictions and more attention to lexical and syntactic cues in HSs and L2 learners, whereas higher use yielded earlier predictions, more attention to lexical cues in L2 learners, and less attention to syntactic cues in HSs and L2 learners. These findings suggest that language proficiency is different from language use and call for additional online studies to determine the individual contributions of language proficiency and use on other types of associations. Using a visual world eye-tracking task, our study fills this gap by investigating whether AoO, language proficiency and language use modulate how HSs and L2 learners form stress-tense suffix associations *within* words.

## 4. The study

Predicting what a person will say facilitates processing efficiency, adaptation, and learning (Kaan and Grüter, 2021). Prediction refers to the unconscious pre-activation of pertinent information before hearing it (Barr, 2008) using multiple linguistic cues (e.g., phonological, morphological, syntactic, and semantic) and non-linguistic cues (e.g., auditory, visual, olfactory). As shown in the background section, most prediction studies investigated AoO and proficiency effects between words (e.g., agreement) in L2 learners using written cues. Studies examining language experience effects on within-word predictions via acoustic cues are rare and show that native speakers use suprasegmental information such as tone or stress to predict word endings, but learners do not always make L2 predictions. There is a growing interest in understanding why this occurs. Is it because the learners began acquiring the L2 later in life? Is it due to insufficient L2 proficiency? Or is it a byproduct of how much the learners currently use the L2?

Bilingual studies on morphophonological associations only investigated the role of L2 proficiency. For instance, higher proficiency was found to facilitate the formation of tone-suffix word associations by L1German-L2Swedish learners (Swedish, but not German, is tonal) in both Swedish verbs (low tones cueing present suffixes and high tones cueing past suffixes; Schremm et al., 2016) and Swedish nouns (low tones cueing singular suffixes and high tones cueing plural suffixes; Gosselke Berthelsen et al., 2018). Instead of using a Swedish proficiency test, Schremm et al. employed the university entry placement test score, and Gosselke Berthelsen et al. used self-ratings. Sagarra and Casillas (2018) administered a Spanish proficiency test and an auditory eye-tracking task to L1English-L2Spanish learners. Advanced, but not beginning, learners predicted stress-tense suffix associations (lexical stress in English and Spanish differ in realization, functional load, and frequency) in Spanish verbs (stressed initial syllables cueing present suffixes and unstressed ones cueing past suffixes). Similar findings were observed in a gating task containing verbs with noise replacing suffixes.

Despite studies showing the effects of AoO and language use on morphosyntactic and phonological processing, the role of these variables on morphophonological prediction within words is unknown. Recent studies with L2 learners who are professional simultaneous interpreters suggest that language use and cognitive resources impact stress-suffix predictions in bilinguals. First, interpreters predicted faster than non-interpreters of the same L2 proficiency level (Lozano-Argüelles et al., 2022), due to their extensive experience making predictions while interpreting. Second, verbal working memory facilitated predictions in monolinguals and interpreter L2 learners, but not non-interpreter L2 learners (Lozano-Argüelles et al., 2022). To determine whether language use also affects stress-suffix predictions in early bilinguals, we recorded the percentage of time participants used Spanish on a weekly basis (see Materials for more information about this measure). Additionally, we compared HSs to L2 learners to advance our understanding of AoO effects on bilingual predictions.

Using an implicit auditory eye-tracking task, we investigated whether verb stress (oxytone, paroxytone), AoO (before, after puberty), language proficiency, and language use modulated how Spanish monolinguals, HSs, and L2 learners formed stress-suffix associations. Regarding stress effects, paroxytones are more common in Spanish words (Morales-Front, 2014), but oxytones are more typical in English disyllabic verbs (Chomsky and Halle, 1968). We expect that stress type will not affect the monolinguals’ predictions due to ceiling effects, and that HSs’ and L2 learners’ dominance in English will produce more fixations on targets with oxytones than paroxytones. Concerning AoO effects, we hypothesize that all groups will predict above chance, based on Sagarra and Casillas’ (2018) findings with monolinguals and non-beginning L2 learners. But we expect the monolinguals to predict earlier than the HSs and L2 learners, following Sagarra et al. (2021). AoO of English was not included because all the HSs began learning English formally at age 5, when they began kindergarten, and because Lloyd-Smith et al. (2019) found that AoO of the majority language did not affect the majority language or the heritage language. Respecting language proficiency effects, we foresee that higher proficiency will increase fixations to target verbs, considering studies with Spanish L2 learners (Sagarra and Casillas, 2018) and Swedish L2 learners (Schremm et al., 2016; Gosselke Berthelsen et al., 2018). As for language use effects, we anticipate that greater language use will produce more fixations to target verbs. This is in line with studies showing that greater language use facilitates morphosyntactic processing (L2 learners: Faretta-Stutenberg and Morgan-Short, 2018; HSs: Foote, 2011; Caffarra et al., 2017; Keating, 2022) and prediction (HSs: Parshina et al., 2022), as well as L2 sound discrimination (Flege and MacKay, 2004; Black et al., 2020), monolingual-like pronunciation in HSs (Lloyd-Smith et al., 2019), and reduced L2 accent (Guion et al., 2000). Lastly, we postulate that language use will have a stronger impact on prediction than language proficiency in both HSs and L2 learners, but particularly in the HSs. This is because language use, but not AoO or L2 proficiency, changes white matter microstructure (Del Maschio et al., 2020), and because language use restructures brain areas associated with language control (DeLuca et al., 2019; Fedeli et al., 2021).

### 4.1. Participants

We collected data from 122 individuals: 30 Spanish monolinguals (M; 22 females), 42 HSs (26 females; with Spanish as the heritage language and English as the majority language), and 50 L2 learners (36 females, L1 English, L2 Spanish). Participants had normal hearing and normal or corrected-to-normal vision and held at least a high school diploma. In addition, they were between 18 and 40 years-old and right-handed. HS data were collected in the U.S. and M and L2 data were collected in Spain. L2 data were gathered in Spain to have L2 learners with high Spanish use comparable to the HSs. The M were born and raised in Madrid, Spain. They spoke English but were not advanced learners, according to self-ratings. Also, they did not speak other languages, and had not lived in a non-Spanish community for more than 2 months. The HS and L2 groups only spoke Spanish and English. HSs were born and raised in the United States, were second generation of immigrants, and had not received formal education in their heritage language, apart from taking Spanish in school. They grew up using Spanish at home and in their neighborhood, and they continued using Spanish in these contexts. Half of them had traveled to their parents’ native country. Approximately 30% of the HSs spoke Spanish with friends, 80% listened to music in Spanish, and 40% watched TV in Spanish. The L2 learners began learning Spanish at least 1 h of class per week in middle school and continued in high school and at the university, and had lived in Madrid an average of 38.29 months (*SD* = 34.12).

The bilingual participants completed language *use* and *proficiency* assessments described in the materials section. The *use* and *proficiency* data were fit to separate Bayesian linear models, in order to assess potential group differences.^1^ The posterior marginal mean difference between groups on both response variables was compared, using a region of practical equivalence (ROPE) of ±0.1. If, for a given measure, the full range of the 95% highest density credible interval (HDI) of the difference estimate fell within the ROPE, the groups were considered to be equivalent. The average Spanish proficiency score was 0.70 (SD = 0.09) for the HS group and 0.71 (SD = 0.14) for the L2 group, the marginal mean difference was 0.02 [−0.03, 0.07], and all the HDI fell within the ROPE. The probability that the effect was positive was 0.77. Regarding Spanish use, the average score was 0.41 (SD = 0.15) for the HS group and 0.38 (SD = 0.16) for the L2 group, the marginal mean difference was −0.02 [−0.09, 0.05], and the HDI fell within the ROPE. The probability that the effect was negative was 0.72. Taken together, we are confident that the groups do not differ in any meaningful way with regard to use or proficiency in Spanish. Table 1 provides descriptive statistics and summarizes the models.

**Table 1:** Language use and proficiency assessments for the HS and L2 bilingual groups.

Metric	HS (*n* = 42)	L2 (*n* = 50)	Contrast	Estimate	ROPE	PD
Proficiency	0.70 (0.09)	0.71 (0.14)	L2 − HS	0.02 [−0.03, 0.07]	1	0.77
Use	0.41 (0.15)	0.38 (0.16)	L2 − HS	−0.02 [−0.09, 0.05]	1	0.72

The table reports the mean and standard deviation, as well as posterior estimates of the marginal mean difference (L2 − HS) and the 95% highest density credible interval (in brackets). The proportion of the posterior density falling within the region of practical equivalence (±0.1) is reported in the ROPE column. The probability that the effect is of the median’s sign is reported in the PD column.

### 4.2. Materials and procedure

Data collection was conducted individually in a single session. Participants completed four tasks. First, the bilingual groups completed a Spanish proficiency test in Qualtrics. The test consisted of a 56-item adapted version of the *Diploma de Español como Lengua Extranjera* (Certificate of Spanish as a Foreign Language) that assessed Spanish grammar and vocabulary knowledge (Sagarra and Herschensohn, 2010). Second, the bilingual groups completed a language background questionnaire with questions regarding age, handedness, languages spoken at home when growing up, AoO, time spent in Spanish-speaking countries, and other languages spoken. Third, the HS and L2 groups filled out a Spanish use questionnaire measuring the percentage of time actively using each language weekly (a combination of input, output, and interaction when talking with friends and family, at work, listening to music, and watching TV).

Lastly, all groups completed an eye-tracking task assessing participants’ abilities to use the stress of a Spanish disyllabic verb’s first syllable to predict the verb’s ending (i.e., the tense suffix) before hearing it. The eye-tracker was an EyeLink 1,000 Plus desktop mount from SR Research (sampling rate: 1 k Hz; spatial resolution of 0.32^o^ horizontal and 0.25^o^ vertical; averaged calibration error: 0.25^o^–0.5^o^). The task was programmed with SR Research’s Experiment Builder software, and the data were extracted with SR Research’s DataViewer software. Tracking was monocular (right eye) and followed cyclopean extraction mode. The velocity threshold (the threshold to consider an eye movement a saccade) was 30°/sec, which is the default for cognitive research in Experiment Builder. Shorter eye movements taking place during fixations (e.g., tremors, drifts, and microsaccades) were considered part of the fixation because numerous studies show that they rarely affect the analysis of higher-level structures such as words or phrases (e.g., Ditchburn, 1980). The monitor was a BenQ XL2420TE display monitor at a resolution of 1,920 × 1,080 pixels, and the headphones were Sol Republic 1601-32.

Participants listened to 100 sentences: four practice sentences, 16 experimental sentences, and 80 fillers. Sentences rather than words were used to imitate naturalistic comprehension and increase ecological validity. The practice sentences appeared always in the same order, and the experimental and filler sentences were distributed into 8 blocks. Each block contained six filler sentences and two experimental sentences, one per condition. Sentences were randomized between blocks and pseudo-randomized within blocks to avoid two consecutive experimental sentences of the same condition. We recorded these sentences using a Fostex DC-R302 digital recorder and a Shure SM10A head-mounted microphone in a Whisper room 6,084 E sound booth at a sampling rate of 44.1 kHz and 16-bit quantization. A Castilian Spanish female speaker unaware of the purpose of the study recorded all the sentences three times in three different pseudo-randomized orders; we chose the clearest pair of the last two repetitions. She used a standard intonation and a consistent rate of 4.37 (*SD* = 0.68) syllables per second and 4.17 (*SD* = 1.14) seconds per sentence. Intensity was normalized to ~75 dB and 100 ms of leading and trailing silence added using Praat (Boersma and Weenink, 2021).

All sentences were grammatical and consisted of 5–14 words. Filler sentences contained anaphora, gender agreement, and idiomatic expressions. Experimental sentences were five words long and followed an SVO word order, with animate noun subjects and inanimate noun objects. Subjects and objects were 2–4 syllables long. Experimental verbs were disyllabic third-person singular regular transitive -ar verbs with a CVC-CV syllabic structure. The mean duration of the verbs was 424 ms (*SD* = 42.22, CI [408.78, 439.22]). Breaking down the verb duration into syllables, the first syllable had a mean duration of 308.38 ms (*SD* = 52.03, CI [284.74, 321, 20]) and the second syllable of 115.63 ms (*SD* = 32.74, CI [108.53, 133.53]). The second syllable disambiguated the tense segmentally. Experimental sentences had two conditions: paroxytone/present and oxytone/preterit (e.g., *El ladrón salta/saltó la valla* “the thief jumps/jumped over the fence”) and only differed in the verb. The visual stimuli consisted of a present and a preterit verb displayed side by side on the screen. Their positions were counterbalanced across participants and trials. We chose words rather than images because (1) it is difficult to illustrate present and past actions, (2) it is uncertain what word participants truly activate when they see an object, and (3) phonological competitor effects are stronger with words than pictures (Huettig and McQueen, 2007; Ito et al., 2017). The written words for the filler sentences consisted of inanimate nouns for the anaphora fillers, descriptive adjectives for the gender agreement fillers, and ending nouns for the idiomatic fillers.

The procedure of the eye-tracking task was as follows: participants were first randomly assigned to one of two versions of the task. Each version contained only one of the two conditions of each verb pair (e.g., if *salta* “s/he jumps” (paroxytone/present) appeared in version 1, then *saltó* “s/he jumped” (oxytone/preterit) appeared in version 2). Both versions had the same number of practice, filler, and experimental trials. Participants rested their heads on a chin rest, completed a 9-point grid calibration task, and received task instructions. Next, participants completed the practice trials, followed by the experimental trials. For each trial, participants saw a + drift correction sign, followed by a 250 ms blank screen, saw two verbs side by side for 1,000 ms, listened to the sentence, and chose the verb on the screen they heard as soon as possible by pressing the left- or right-shift key. Upon pressing either key, a rectangle appeared around the selected verb. Participants did not receive feedback after completing the task. We set up response recording to register only when the keypress happened at or after the onset of the verb. Key presses did not stop the sound file. After each sentence, a blank screen appeared for 500 ms, and the next trial began. After the eye-tracking task, participants completed a test assessing their knowledge of the meaning of the experimental verbs (e.g., to know that *salta* means to jump) and the tense suffixes (e.g., to know that *salta* is present). Participants saw a list containing the experimental Spanish verbs and a list containing English verbs. Their task was to match each Spanish verb with the correct English translation.

### 4.3. Statistical analyses

We fit a series of Bayesian regression models to examine the time course data. The primary model was a Generalized Additive Mixed Model (GAMM, Winter and Wieling, 2016; Sóskuthy, 2017). GAMMs are useful for scrutinizing non-linear data, such as that typically associated with eye-tracking.^2^ In subsequent analyses, we summarized the posterior predictive distribution to make inferences about the relationships between speaker groups, lexical stress, language proficiency, and language use. Given the distinct nature of some of these analyses, we provide a brief description of the statistical approach at the beginning of each subsection. For all models, we employed regularizing, weakly informative priors (Gelman et al., 2017).^3^ In most cases, we used the following formula to establish a region of practical equivalence (ROPE) around a point null value (see Kruschke, 2018):

$$ROPE=\frac{\mu_{1}-\mu_{2}}{\sqrt{\frac{\sigma_{1}^{2}+\sigma_{2}^{2}}{2}}}$$

We report mean posterior point estimates for parameters of interest, along with the 95% highest density credible interval (HDI), the percent of the region of the HDI contained within the ROPE, and the probability of direction for each effect (PD). For statistical inferences, we focus on estimation rather than decision-making rules, though, generally, a posterior distribution for a parameter β in which 95% of the HDI falls outside the ROPE and a high PD (i.e., values close to 1) are taken as compelling evidence for a given effect. We conduct all analyses using R (version 4.2.1) and fit all models using the probabilistic programming language *stan* via the R package *brms* (Bürkner, 2017, 2018).

## 5. Results

The analyses are divided into three sections. First, we describe the trajectories of the time course. Then, we evaluate suffix prediction at the target syllable offset. Lastly, we consider the effects of language use and proficiency.

### 5.1. The time course of morphological processing

**Figure 1:**
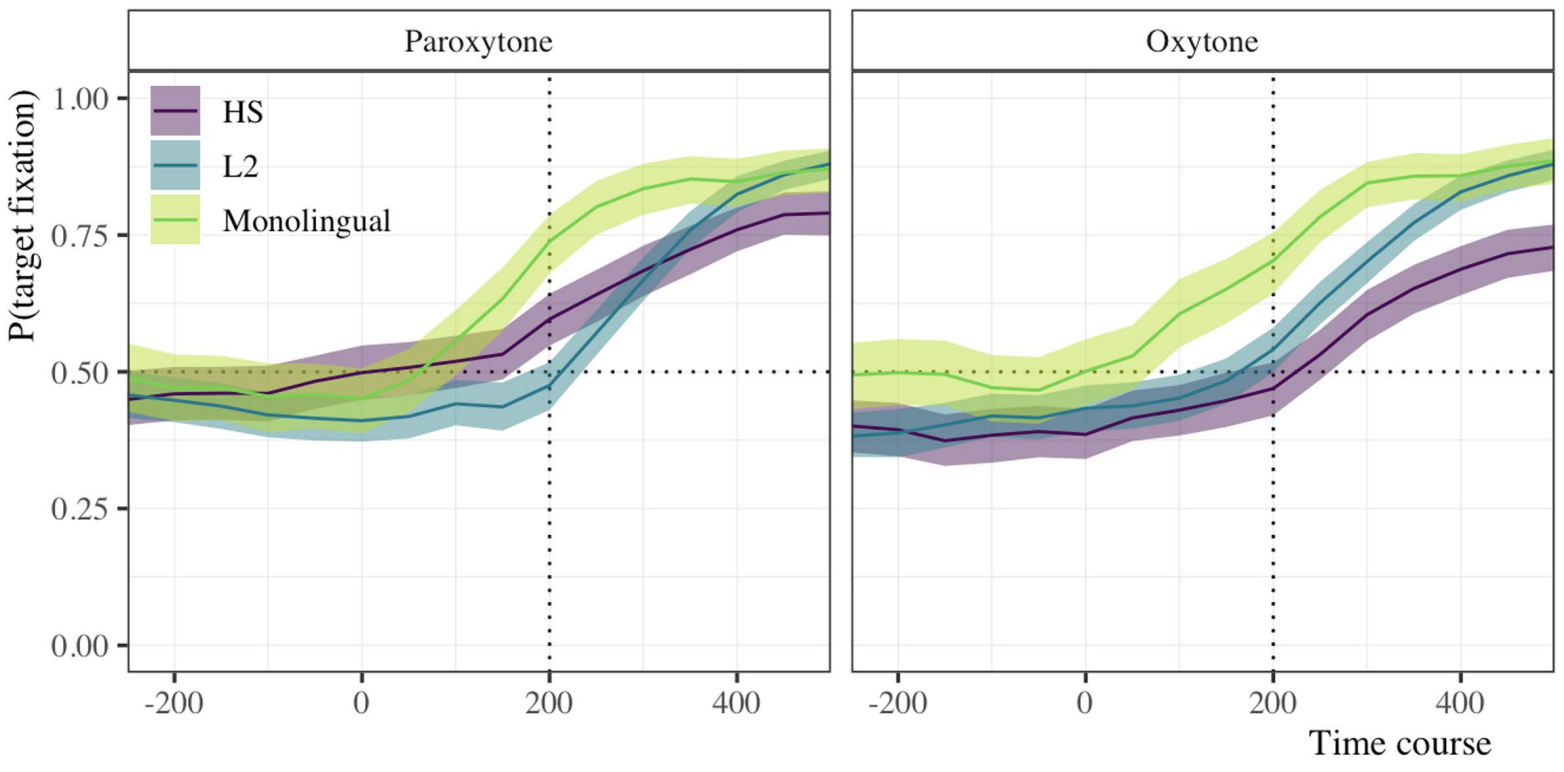
Time course of raw target fixation data as a function of stress condition (paroxytone, oxytone) for monolingual, HS, and L2 groups. Transparent ribbons represent 99% bootstrapped confidence intervals.

Our analysis of the time course data from the eye-tracking task models measures how the probability of fixating on target items changes over time and under different suprasegmental conditions. We down-sampled the data to bins of 50 ms which were centered at the offset of the first syllable of target items. The time course of fixation used for analysis ranged from 200 ms before target syllable offset to 600 ms after. We chose this window because it captures the portion of the time course in which target fixations began to steadily increase from chance. Figure 1 illustrates the trajectories of the monolinguals, HSs, and L2 learners as a function of lexical stress. In both panels, we see that the probability of fixating on the target hovers around 0.5 and begins to increase as time increases. Notably, we also observe that the lines are not overlapping. The monolingual group begins to fixate on the target earlier in the time course in both paroxytones and oxytones. Essentially, the HSs and L2 groups are phase shifted to the right, representing later target fixations.

**Figure 2:**
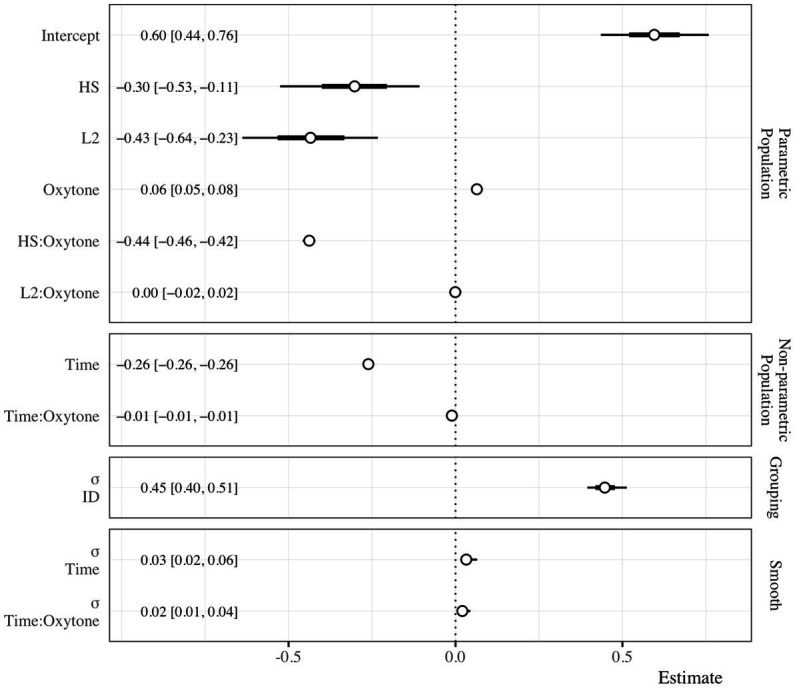
Forest plot of the omnibus GAMM. The horizontal axis represents the models estimates in log-odds. The vertical axis lists the terms estimated in the model. The points illustrate the posterior mean along with the 66% and 95% HDI. The vertical faceting separates the estimates into parametric and non-parametric population-level effects, group-level effects, and smooth terms.

Given the binary nature of the dependent variable (“i.e., fixations on the target word vs. elsewhere), we assumed that the likelihood was going to be binomially distributed. The model assessed target fixations as a function of the parametric terms *group* (monolingual, HSs, L2), *stress* (paroxytone, oxytone), and a nonlinear function of time. Both *group* and *stress* were set as ordered variables with monolinguals and paroxytones coded as “0.” We implemented cubic regression splines with four basis knots: (a) as a reference smooth to time, (b) as a difference smooth to time conditioned on stress, and (c) as a random smooth for each participant conditioned on time. Thus, the trajectory of the monolinguals’ target fixations to paroxytone words (e.g., *CANta*) served as the baseline, and we could compare it to the trajectories of the other groups. The forest plot in Figure 2 illustrates the model summary (see Supplementary material for the complete summary in table form).

**Figure 3:**
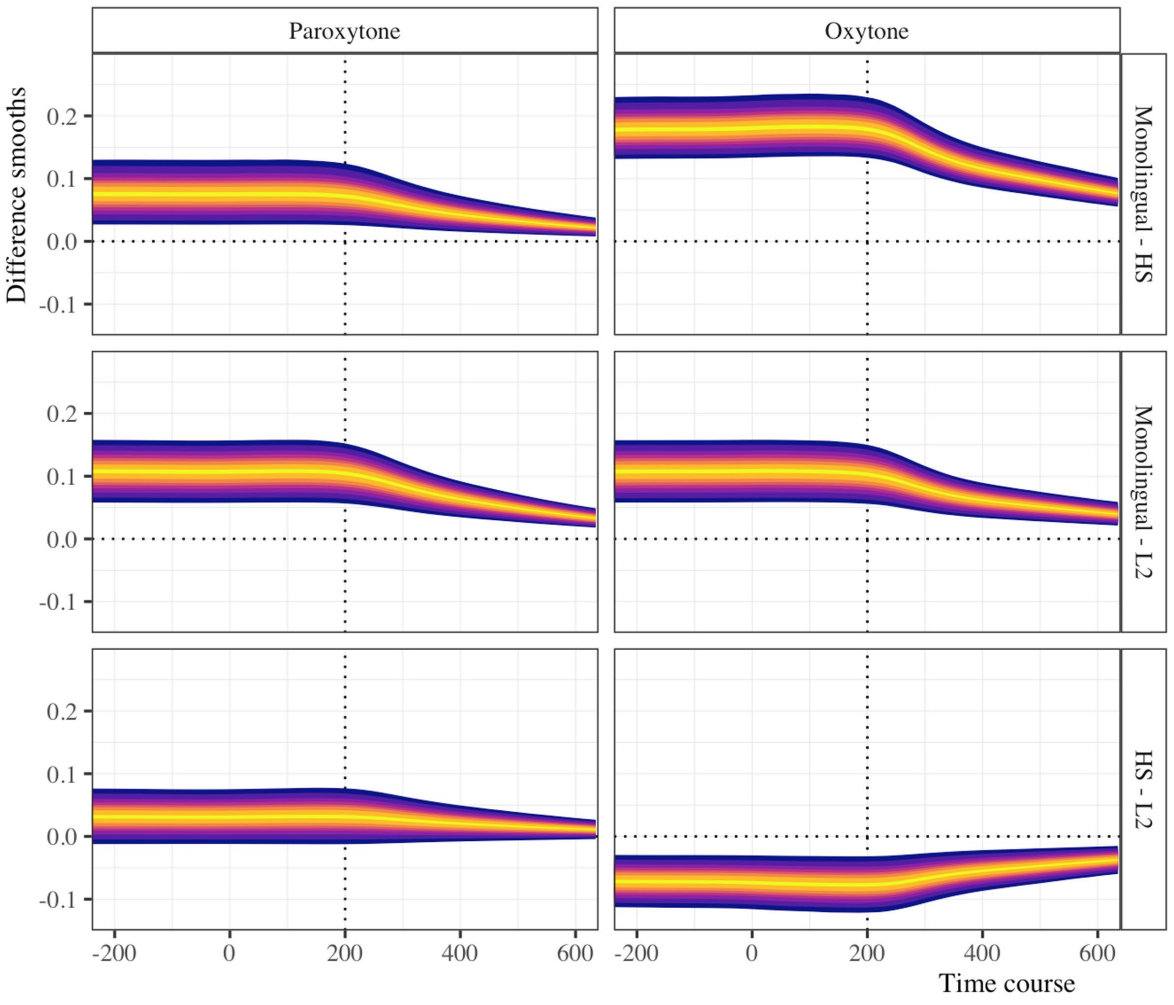
Pairwise difference smooths for paroxytone and oxytone items. From dark to light, the colors represent 95%, 80%, 70%, 60%, 50%, 35%, and 10% highest density credible intervals.

To quantify and assess the between-group differences over time, we used the posterior predictive distribution to calculate posterior pairwise difference smooths. Figure 3 illustrates these pairwise comparisons over the time course in the probability space. Overall, the analysis shows that the monolingual group fixates on targets earlier than the HS and L2 groups in both stress conditions over the time-window we selected. The HS-L2 comparison suggests that the HS group fixates on targets slightly more and earlier in paroxytone condition, but the opposite is true in the oxytone condition.

### 5.2. Prediction at target offset

**Figure 4:**
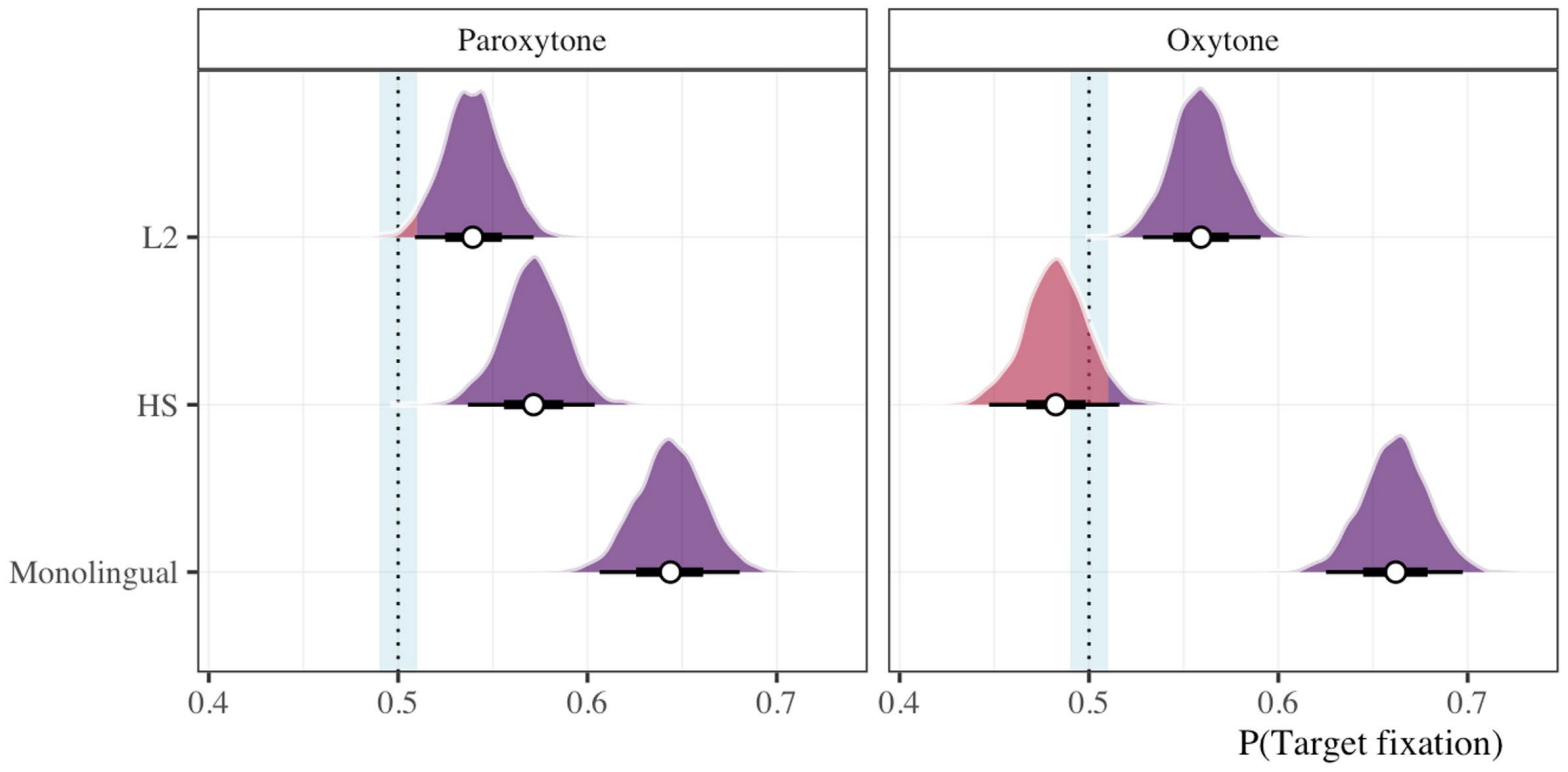
Proportion of target fixations 200 ms after the offset of 1st syllable for monolingual, HS, and L2 groups in paroxytone (CANto) and oxytone (canTO) conditions. The vertical dotted line marks chance (50%) surrounded by a ± 1% region of practical equivalence (ROPE). The density mass of a posterior distribution that falls below the upper bound of the ROPE is displayed in red and values above this threshold are purple.

In order to assess the participants’ ability to predict suffixes, we used the posterior predictive distribution of target fixations 200 ms after the target syllable offset (i.e., the minimum time necessary to plan and launch a saccade, see Fischer, 1992). We considered that the probability that target fixation was greater than chance at this time point for each group in each stress condition and implemented a ROPE of 0.01 around a point null, chance value of 0.5. Figure 4 illustrates posterior distributions of target fixations.

All groups fixated on targets above chance 200 ms after the target syllable offset with the exception of the HS group in the oxytone condition (β = 0.48, HDI = [0.45, 0.52], ROPE = 0.97, PD = 0.85). Approximately 97% of the HDI fell below the upper bound of the ROPE and there is an 85% chance that the estimate is below 0.5. Additionally, a small portion of the posterior probability mass of the L2 group in the paroxytone condition fell within the ROPE (β = 0.54, HDI = [0.51, 0.57], ROPE = 0.01, PD = 1), though, given the model, the data and our prior assumptions, the effect is nearly certain to be above 0.5.

**Figure 5:**
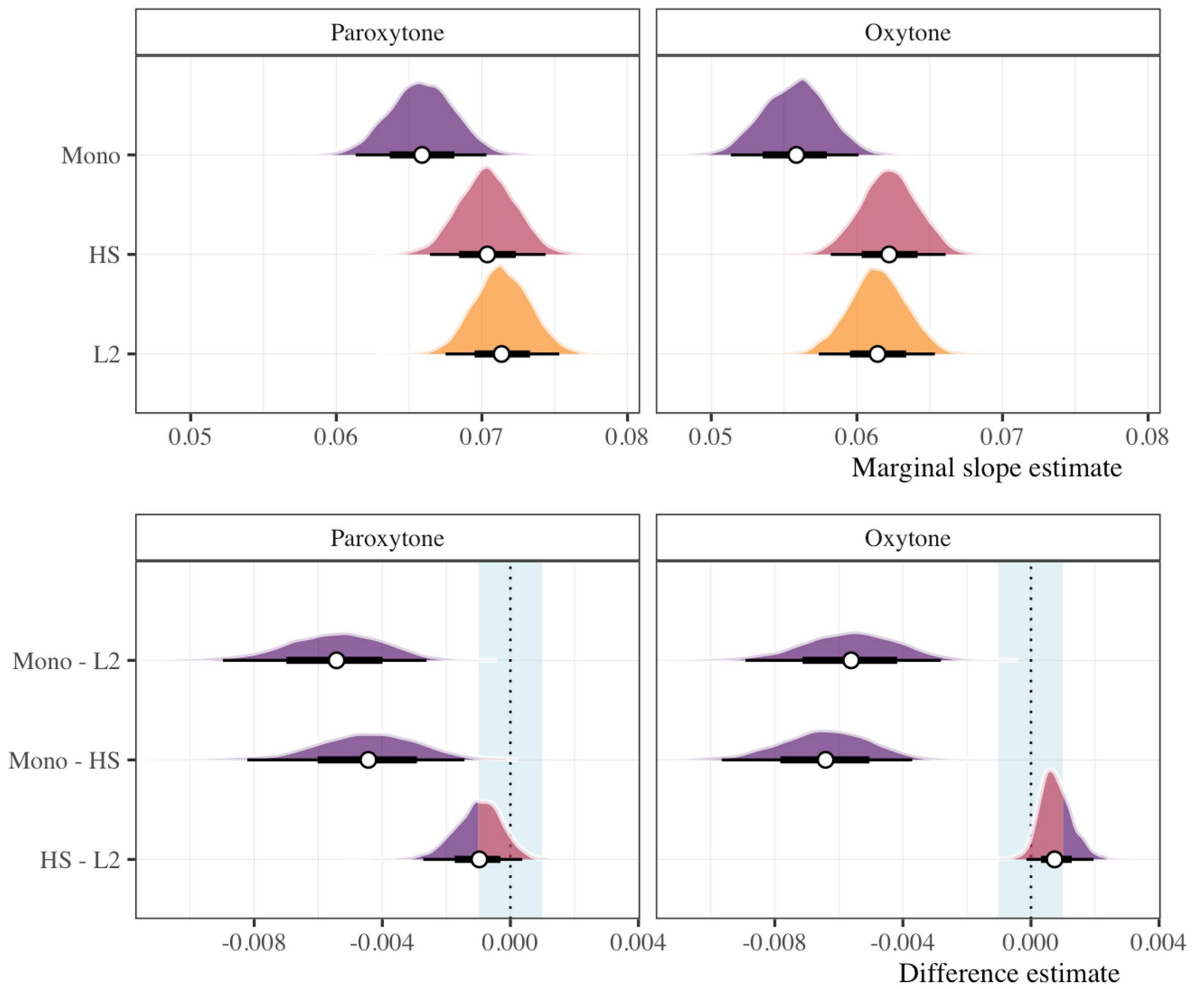
Marginal slope estimates (top) and pairwise difference estimates (bottom) for monolingual, HS, and L2 groups 200 ms after the target syllable offset in paroxytone (CANto) and oxytone (canTO) conditions. Points represent posterior means along with the 66 and 95% HDI. In the bottom panels, the vertical blue rectangle illustrates a ROPE of ±0.001. Posterior mass falling within the ROPE is depicted in red and values outside the ROPE are in purple.

Subsequently, we assessed the rate of target fixations at the same time point (i.e., 200 ms after the offset of the target syllable). While the previous assessment evaluates *if* participants fixate on targets before hearing a critical suffix, this analysis sheds light on *how fast* target fixations occur by calculating the partial derivative (i.e., slope) of the trajectory at this time point. The top panels of Figure 5 show the marginal slope estimates for each group for paroxytone and oxytone words. The bottom panels of Figure 5 provide pairwise group comparisons in each condition. The monolingual group demonstrates a slower rate of target fixation (i.e., a less steep slope) than the HS group for paroxytones (β = −0.004, HDI = [−0.008, −0.001], ROPE = 0, PD = 0.999) and oxytones (β = −0.006, HDI = [−0.010, −0.004], ROPE = 0, PD = 1). This is also the case when compared with L2 learners (paroxytones: β = −0.006, HDI = [−0.009, −0.003], ROPE = 0, PD = 1; oxytones: β = −0.006, HDI = [−0.009, −0.003], ROPE = 0, PD = 1). Upon evaluating the HS and L2 groups, we do not find compelling evidence that either group has a faster rate of target fixation. In the paroxytone condition, the L2 group might be slightly faster, but nearly half the HDI fell within the ROPE (β = −0.001, HDI = [−0.003, 0.000], ROPE = 0.514, PD = 0.922). In the oxytone condition the opposite is true. That is, the L2 group may have been slightly slower, but, again, a large portion of the HDI fell within the ROPE (β = 0.001, HDI = [0.000, 0.002], ROPE = 0.697, PD = 0.957). Taken together, we do not believe there is compelling evidence that the rate of target fixation differs between the HS and L2 groups. Additional plots and a table summary are provided in the Supplementary material.

### 5.3. Proficiency and use

To assess the effects of language proficiency and use, we took the subset of the HS and L2 data from the time bin that corresponded with 200 ms after the offset of the initial syllable in the target items. We calculated the proportion of target fixations for each participant, in each condition and submitted these proportions to a zero-inflated beta regression model.^4^ The outcome was modeled as a function of group (HS, L2), stress (paroxytone, oxytone), use, and proficiency. Group and stress predictors were sum coded (−1, 1) and the continuous predictors were standardized (i.e., converted to z-scores), thus the model intercept provided an estimate of target fixation marginalizing over *group* and *stress*, with *use* and *proficiency* equal to 0 (i.e., at the unstandardized mean). The model included all two-way interactions as well as the group by use by proficiency three-way interaction. We included a group-level effect for participants with a varying slope for stress. A full description of the model specification and priors is available in the Supplementary material.

**Figure 6:**
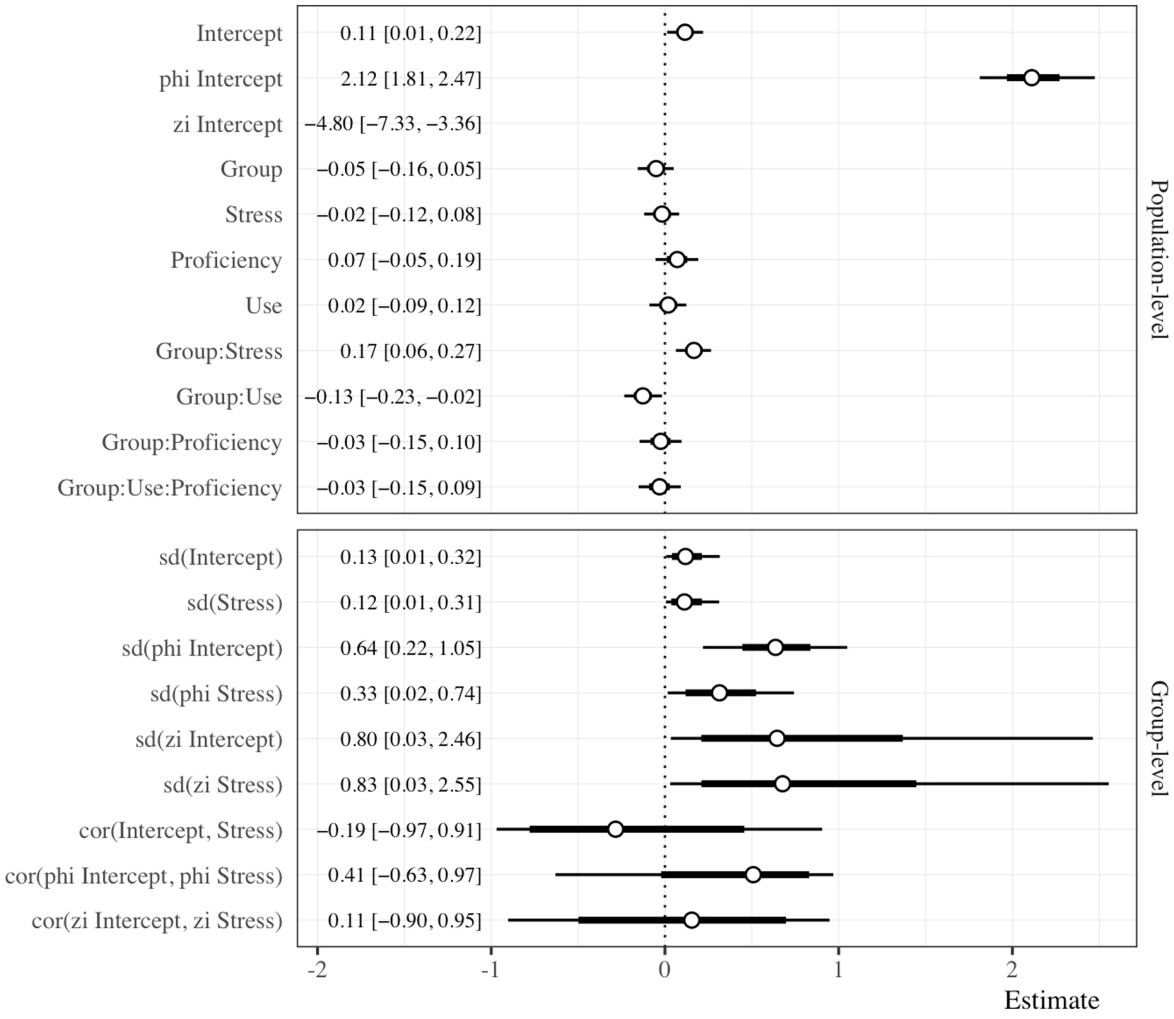
Forest plot of the zero-inflated beta regression. The horizontal axis represents the models estimates in log-odds. The vertical axis lists the terms estimated in the model. The points illustrate the posterior mean along with the 66% and 95% HDI. The vertical faceting separates the estimates into population-level and group-level effects.

The overall probability of fixating on a target was approximately 0.53 (Intercept: β = 0.11, HDI = [0.01, 0.22], ROPE = 0.39, PD = 0.99). There was no main effect for group (β = −0.05, HDI = [−0.16, 0.05], ROPE = 0.83, PD = 0.83), nor stress (β = −0.02, HDI = [−0.12, 0.08], ROPE = 0.97, PD = 0.63), though the two predictors did interact (β = 0.17, HDI = [0.06, 0.27], ROPE = 0.08, PD = 1). Holding proficiency and use constant at their mean, the HS group fixated on targets at a higher rate in the paroxytone condition (β = 0.57, HDI = [0.45, 0.69]) than in the oxytone condition (β = 0.48, HDI = [0.38, 0.58]). The opposite was true for the L2 group (paroxytone: β = 0.54, HDI = [0.42, 0.64]; oxytone: β = 0.47, HDI = [0.35, 0.61]). The forest plot provided in Figure 6 summarizes the model. A model summary table is available in the beta regression subsection of the Supplementary material.

**Figure 7:**
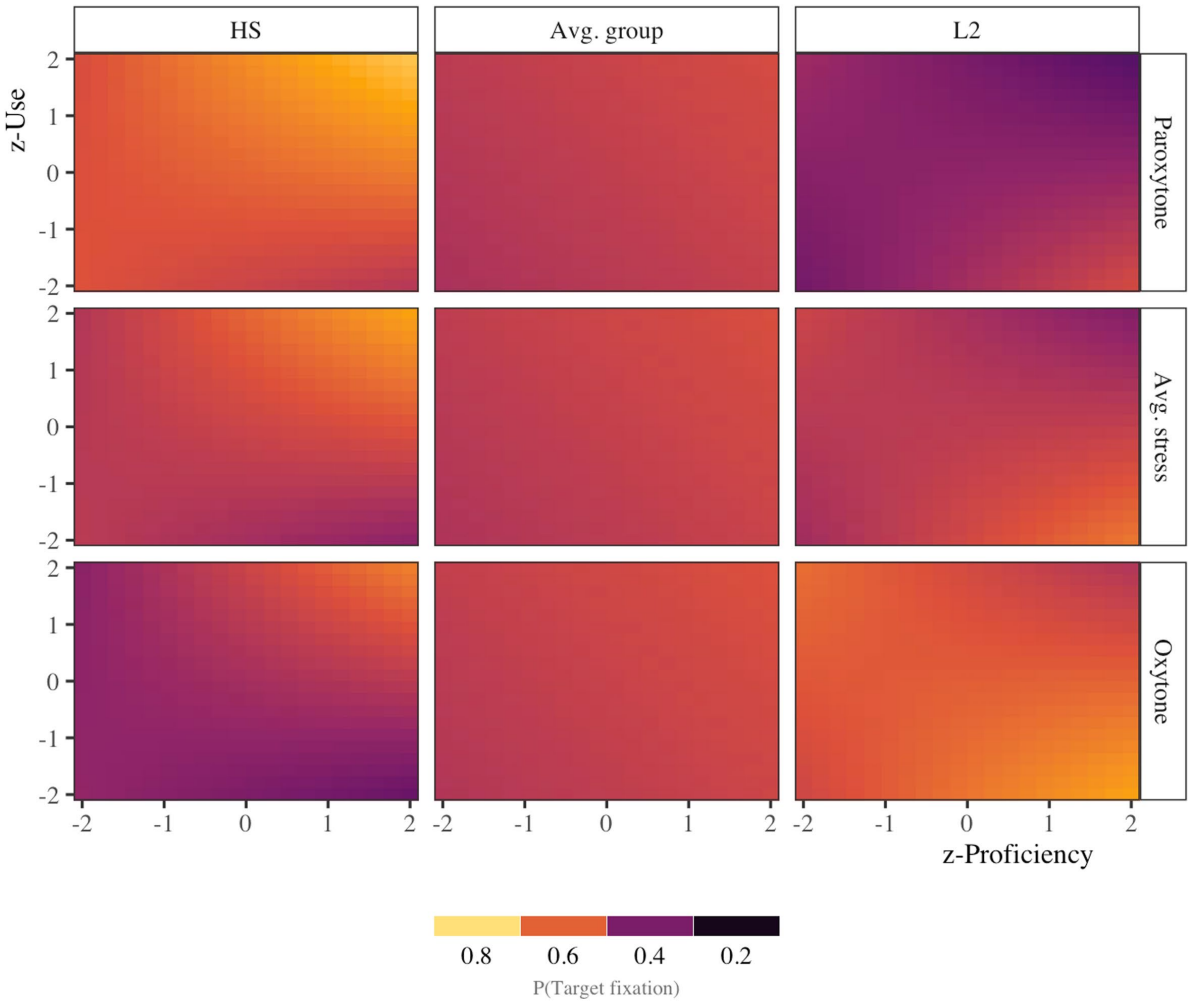
Heatmap of target fixations 200 ms after target syllable offset. The heatmap illustrates the marginal effects of normalized proficiency and use scores. The top rows illustrate model estimates for the paroxytone condition, the middle rows marginalize over stress conditions, and the bottom rows represent model estimates for the oxytone condition. Moving from left to right, the first column provides estimates for the HS group, the middle column marginalizes over groups, and the rightmost column presents estimates for the L2 group. The vertical and horizontal axis display standardized language use and language proficiency (±2 SD), respectively.

There was also evidence of a group × use interaction (β = −0.13, HDI = [−0.23, −0.02], ROPE = 0.3, PD = 0.99). Although approximately 30% of the HDI fell within the ROPE, the model, and our prior assumptions, we are 99% certain that the interaction effect is negative. Figure 7 provides a heat map that illustrates the relationship between proficiency, use, and stress in the bilingual groups. For the HS group, one observes higher target fixations (lighter colors) in the upper right-hand corners of each panel. That is to say, HSs fixated more on targets higher levels of use and proficiency, particularly in the oxytone condition. Target fixation was higher, nearly across the board, in the paroxytone condition. For the L2 group, on the other hand, one observes a higher propensity to fixate more on targets in the lower right-hand corners of each panel (lighter colors), when proficiency is higher, but not necessarily language use. Unlike the HS group, the L2 group seldom predicted in the paroxytone condition (upper right panel).

## 6. Discussion

We investigated whether AoO, language proficiency, and language use influenced how Spanish HSs and L2 learners form stress-tense suffix associations in Spanish disyllabic verbs, using an auditory eye-tracking task. Regarding the effects of stress and AoO, holding proficiency and use means constant, all groups fixated on target verbs above chance before hearing the syllable with the suffix in all conditions. The only exception occurred with the HS group in the oxytone condition. Furthermore, HSs predicted less with oxytones, whereas L2 learners predicted less with paroxytones. Monolinguals fixated on targets more and earlier, but at a slower rate, than bilinguals in all conditions, and HSs more and earlier than L2 learners in paroxytones. However, HSs predicted later than L2 learners with oxytones. With respect to proficiency and use, HSs with higher proficiency and greater language use fixated on target oxytones more. Yet, while greater use was more important than higher proficiency for HSs, L2 learners with higher proficiency fixated on target paroxytones more, and the amount of use did not matter. These results show that L2 learners can acquire stress-suffix associations absent in their L1 after puberty, and that their ability depends on their L2 proficiency level rather than their AoO or L2 use. Next, we discuss the relevance of our findings with respect to stress type (oxytone, paroxytone), AoO (before, after puberty), language proficiency, and language use.

### 6.1. The effects of stress

Paroxytone words have the stress on the penultimate syllable (e.g., *SALta* “she/he jumps”), whereas oxytone words have it on the last syllable (e.g., *salTÓ* “she/he jumped”). The majority of Spanish words (Morales-Front, 2014) and English words (Kelly and Bock, 1988) are paroxytone. However, in English, oxytones and paroxytones are equally frequent in disyllabic uninflected words (Clopper, 2002), and oxytones are more frequent than paroxytones in disyllabic verbs (Chomsky and Halle, 1968). Relevant to our study, in Spanish, third person singular regular verbs are more frequent in present tense (paroxytone; 30,667/1,000,000) than preterit tense (oxytone; 12,030/1,000,000; CORPES, Real Academia Española). Furthermore, Spanish and English have contrastive stress, but suprasegmental cues have a greater functional load in Spanish than in English. Considering these data, we hypothesized that the monolinguals would predict regardless of stress type, that the HSs would predict more than the L2 learners, and that the HSs and L2 learners would predict more with oxytones than paroxytones because oxytones are more common in disyllabic verbs in their dominant language, English. The results of the tasks confirmed our hypothesis with the monolinguals. This group used stress to predict suffixes before hearing them above chance with both paroxytones and oxytones. These results are in line with studies showing that Swedish speakers use tone to predict number (singular vs. plural; Roll et al., 2010, 2013; Söderström et al., 2016) and tense (present vs. past; Roll, 2015; Söderström et al., 2016), and that Spanish speakers use stress to predict tense (present vs. past) with both paroxytones and oxytones (Sagarra and Casillas, 2018).

HS and L2 data did not support our hypothesis. Holding proficiency and use means constant, the L2 learners predicted above chance with both paroxytones and oxytones, in line with Sagarra and Casillas (2018). However, the HSs only predicted above chance with paroxytones, the L2 learners predicted more and earlier than the HSs with oxytones, and the HSs predicted more and earlier than the L2 learners with paroxytones. The differences between the HSs and the L2 learners can be explained by HSs’ early AoO, more years of exposure to Spanish, or higher number of lexical competitors. First, the difference between HSs and L2 learners cannot be linked to AoO because L2 learners predicted above chance in both the English-preferred condition (oxytones) and the Spanish-preferred condition (paroxytones). Second, the difference is likely not due to HSs’ longer accumulated exposure to Spanish paroxytones (lexical frequency), the preferred condition in Spanish, because the HSs had trouble with oxytones, the preferred pattern in their dominant language, and because Sagarra et al. (under review) found no differences between English and Mandarin learners of Spanish, although lexical tone T4—which resembles paroxytones—is more frequent in Mandarin, and suprasegmentals have a higher functional load in Mandarin and Spanish than in English. Third, we attribute the differences between HSs and L2 learners to their current use of Spanish, in particular to lexical competition due to co-activation. The learners have a native lexicon and later-acquired, more fragmented L2 lexicon that makes the L1 lexicon dominate. This explains the learners’ stronger and faster activation of oxytones, the preferred pattern in English. In contrast, the HSs have two L1 lexica that rapidly activate when hearing words. This produces higher competition in oxytones because English is their dominant language and English has more oxytone than paroxytone candidates, making it harder to use oxytone predictors (predictions are stronger for word beginnings that evoke few lexical competitors, e.g., Söderström et al., 2016). Our findings support theoretical models explaining HSs’ variability and divergence from monolinguals in terms of lexical competition due to co-activation (e.g., Hatzidaki et al., 2011; Giezen and Emmorey, 2016). Our results also show that such competition exerts a greater influence on how HSs access words than lexical frequency, contrary to theoretical accounts proposing that lexical frequency offsets lexical competition (e.g., Hur et al., 2020; Perez-Cortes, 2020). Importantly, these studies employed offline tasks and examined morphosyntax (grammatical gender agreement) and syntax (mood). Finally, our results are in line with recent neurocognitive studies showing that higher language use increases functional brain connectivity and inhibitory control (see Pereira Soares, 2022, for a review).

One may argue that, because the participants saw a specific present-preterit verb pair before listening to each sentence, they focused on calibrating the frequency of the verbs on the screen to prioritize the most frequent verb pair and they ignored other lexical competitors. To explore this possibility, we calculated the lexical frequencies of the experimental verbs with the *LEXESP* dictionary of frequencies (Sebastián-Gallés, 2000). The experimental paroxytone verbs have a higher lexical frequency than their oxytone counterparts: 131.44 for paroxytones vs. only 44.94 for oxytones. If lexical frequency of the experimental verbs drives prediction, we would expect both bilingual groups to predict more and earlier with paroxytones than oxytones. However, (a) the L2 learners predicted equally with paroxytones and oxytones, (b) the L2 learners predicted oxytones more and earlier than the HSs, and (c) the HSs predicted paroxytones more and earlier than the L2 learners. These three findings demonstrate that the groups did not rely on the experimental verbs’ lexical frequency. Although the task reduced the lexical cohort to two members, the experiment tapped into more automatic processes of lexical access, making participants considered additional lexical competitors.

Lexical frequency is relevant once a prediction has been made (token frequency), but phonotactic frequency determines what competitors are considered as prediction unfolds (type frequency). Sagarra and Casillas (in progress) investigated the role of both phonotactic and lexical frequency on suprasegmentals (oxytone, paroxytone stress) and segmentals (CVC, CV syllabic structure) in advanced HSs and L2 learners. Eye-tracking data showed that higher phonotactic frequency increased fixations on targets in the HSs in all conditions, but not the L2 learners; also, lexical frequency did not affect HSs or L2 predictions. HS, but not L2 learners, consider phonotactic frequency when predicting, due to HSs’ longer experience with the target language.

Taken together, the findings of Sagarra and Casillas (in progress) and of the current study indicate that HSs’ lexical access depends on: (1) number of lexical competitors (HSs consider more competitors than monolinguals and L2 learners because HSs activate two L1 lexica); and (b) type frequency (higher phonotactic frequency affects HSs, but not L2 learners). Token frequency (lexical frequency) may also influence prediction (more frequent words tend to be more strongly activated, e.g., Roll et al., 2013), but cohort size seems to be the most important factor for HSs. Our data also demonstrate that we store suprasegmental information as we access words during comprehension and production, and we prioritize this information over semantic information when we start listening to a word to anticipate the ending.

### 6.2. The effects of AoO

To determine whether adults are able to make L2 stress-suffix associations absent in their L1 if they begin learning the L2 after puberty, we compared adult HSs and adult L2 learners with the same Spanish proficiency level and current use of Spanish. The results support our hypothesis that monolinguals, HSs, and L2 learners would predict above chance. Our L2 data are in consonance with studies indicating that non-beginning L2 learners predict tense suffixes using tone (Schremm et al., 2016) and stress information (Sagarra and Casillas, 2018), and number suffixes using tone information (Gosselke Berthelsen et al., 2018). Although there are no studies on lexical prediction with HSs, our HS data are consistent with studies suggesting that HSs make both syntactic predictions (Sekerina and Trueswell, 2011; Jegerski and Sekerina, 2020) and morphosyntactic predictions (Fuchs, 2021; Sagarra et al., 2021).

Without the HSs, our results could be erroneously interpreted as monolinguals predicting more and earlier than L2 learners due to the learners’ late AoO. The presence of a HS group was necessary to discard this supposition in four ways. First, the monolinguals predicted more and earlier than the HSs, even though both groups began acquiring Spanish at birth. Second, the L2 learners predicted more and earlier with oxytones than the HSs (and the opposite pattern applies to paroxytones), even though the learners began learning Spanish years later than the HSs. Third, although AoO is later in the learners than the HSs and similar in the monolinguals and the HSs, only the monolinguals and the L2 learners predicted above chance with oxytones. Finally, the bilingual groups predicted faster than the monolinguals, and all the bilingual groups predicted at equal speed, regardless of the AoO differences between both bilingual groups. Collectively, these findings suggest that prediction differences between monolinguals and L2 learners and between HSs and L2 learners may not be due to AoO but to differences in the amount and type of experience each group has had with Spanish.

The absence of AoO effects is on par with studies showing no differences between HSs and L2 learners using self-paced reading methodology (Foote, 2011; Rodríguez and Reglero, 2015), eye-tracking technique (written: Sagarra and Rodríguez, 2022; auditory: Sagarra et al., 2021), and ERPs (Wartenburger et al., 2003; Martohardjono et al., 2017). Singleton and Leśniewska (2021) argued that the critical period hypothesis is unfalsifiable and therefore irrelevant, because separating early and late bilinguals is fictional, considering the enormous degree of variability of individual language experiences in each of these two groups. These proposals are timely, given recent neurocognitive studies demonstrating how white matter microstructure changes with later AoO (Nichols and Joanisse, 2016; DeLuca et al., 2019), as well as with greater L2 use (Del Maschio et al., 2020; but see studies showing similarity in the brains of early (0–3 years) bilinguals and monolinguals, but increased cortical thickness in L2 learners). Finally, both AoO and L2 use influence brain areas related to cognitive control, but only L2 use affects areas normally activated during overall language comprehension and production (Fedeli et al., 2021). Our findings suggest that a person’s ability to use suprasegmental information with acoustic realization different from the L1 is intact after puberty. Ultimately, the determining factor in successful learning is the amount of experience with the target language.

### 6.3. The effects of language proficiency

We measured proficiency with an adapted version of the DELE test, which assessed grammatical and vocabulary knowledge of Spanish. Our hypothesis that higher proficiency in Spanish would yield more fixations on targets was partially supported. The data revealed that proficiency interacted with group and stress: higher proficiency increased fixations on targets in HSs with paroxytones and in L2 learners with oxytones. Proficiency did not affect L2 learners’ fixations on targets in paroxytones or HSs’ fixation on oxytones. This makes sense because oxytones are the preferred condition for the L2 learners, whereas paroxytones are the preferred condition for the HSs. The beneficial effects of higher proficiency on L2 learners are consistent with studies with non-beginning learners forming L2 morphophonological associations (tone-suffix: Schremm et al., 2016; Gosselke Berthelsen et al., 2018; stress-suffix: Sagarra and Casillas, 2018). In contrast with our findings, Sagarra and Casillas also observed proficiency effects with oxytones. We speculate that differences in statistical analyses (GCAs vs. Bayesian) can explain the difference.

Overall, our results align with L2 and HS online studies that show positive outcomes stemming from higher language proficiency. Behavioral L2 studies revealed that higher L2 proficiency facilitated L2 prediction based on morphosyntactic associations (e.g., Lew-Williams and Fernald, 2010; Dussias et al., 2013; Sagarra et al., 2021; Henry et al., 2022; see Ito and Pickering, 2021, for a review; and see Mitsugi, 2020, for lack of proficiency effects), phonosemantic associations (Perdomo and Kaan, 2021, for bin 5), and morphophonological associations (Sagarra and Casillas, 2018). Higher L2 proficiency also benefited L2 morphosyntactic processing (see Kirova and Camacho, 2021, for a review), as well as L2 morphological processing (Kimppa et al., 2019), L2 word activation (Berghoff et al., 2021), and L2 phonological processing (Jun and Oh, 2000; White et al., 2015; Konishi et al., 2018; Maddah and Reiterer, 2018). Neurocognitive L2 studies indicated that higher L2 proficiency facilitated L2 morphosyntactic processing (see Alemán Bañón et al., 2018, for a review) and shaped the brain (Pliatsikas et al., 2020), allowing learners to activate the same brain areas as monolinguals (Vingerhoets et al., 2003).

HS studies produced mixed findings. Behavioral HS studies examining L2 morphosyntactic prediction indicated that higher proficiency in the heritage language yielded more and faster fixations on targets (Sagarra et al., 2021). On the other hand, others showed no proficiency or AoO effects (Sagarra and Rodríguez, 2022). This difference may be attributed to Sagarra and Rodríguez’s employment of a written task (Sagarra and Varela used an auditory task) and the type of grammatical structure (adjacent subject-verb number agreement, acquired early, vs. grammatical gender agreement, acquired late). Additionally, while several neurocognitive HS studies revealed beneficial proficiency effects on grammatical processing (Bice and Kroll, 2021), others did not demonstrate any proficiency effects on grammatical processing (Wartenburger et al., 2003, found that AoO, but not proficiency, was related to grammatical processing). Certain studies also merged proficiency and AoO effects in a “multilingual experience” composite score and were therefore unable to disentangle the effects of each (Hervais-Adelman et al., 2018).

### 6.4. The effects of language use

In our study, language use refers to the percentage of time actively using Spanish, and includes input, output, and interaction. Our findings partially supported our hypothesis that greater language use would increase fixations on targets in HSs and L2 learners. Higher language use increased fixations on oxytone targets in HSs, but language use did not influence any other condition or group. The benefits of greater language use on HSs concur with studies that demonstrated how greater use of the heritage language produced more and earlier fixations on targets in morphosyntactic predictions (Sagarra et al., 2021) and increased sensitivity to morphosyntactic violations (Caffarra et al., 2017). Our findings also align with HS studies showing: comparable sensitivity to morphosyntactic violations in HSs and bilingual native speakers raised abroad (Foote, 2011); comparable sensitivity to syntactic violations in HSs and L2 learners raised abroad (Jegerski and Sekerina, 2020); greater sensitivity to morphosyntactic violations in sequential than simultaneous bilinguals (the former use their heritage language more; Keating, 2022); slower syntactic processing in HSs than monolinguals (Sekerina and Trueswell, 2011); and greater sensitivity to morphosyntactic violations with higher literacy experience (Parshina et al., 2022).

So, why did the L2 learners not benefit from using Spanish more daily? Possible explanations are: the L2 learners had less lexical competitors because their Spanish is fragmented; the HSs had been exposed to Spanish longer than the L2 learners; the HSs had mostly acquired Spanish by actively using it, whereas the L2 learners had mostly acquired Spanish in classroom settings. Future online studies comparing HSs varying in their degree of exposure and current use of their heritage language will shed light on this question (in progress).

### 6.5. The relationship between language proficiency and use

The inclusion of language proficiency and use measures within the same sample pool provided us with the unique opportunity to examine how much weight each of these variables exerts on both early and late bilinguals’ processing. Our hypothesis that language use would have a stronger influence than language proficiency was supported for the HSs and rejected for the L2 learners. In effect, language use accounted for prediction in the HS group more than proficiency. With oxytones, the HSs showed maximum prediction with [+proficiency, +use], medium prediction with [−proficiency, +use], low prediction with [+proficiency, −use], and minimum prediction with [−proficiency, −use]. As previously mentioned, neither proficiency nor use affected HSs’ prediction with paroxytones, their preferred condition. On the contrary, higher proficiency facilitated L2 learners’ predictions with paroxytones, regardless of amount of L2 use. Neither proficiency nor use affected L2 predictions with oxytones, their preferred condition. As stated earlier, we attribute the absence of language use effects in the L2 learners to less lexical competitors and to more years of learning confined to the classroom. In a classroom context, learners normally learn *about* Spanish (grammar, vocabulary) and devote a less-than-ideal amount of time to actively *using* Spanish. Teachers typically do not cover stress-tense suffix associations in class, so learners need to learn these associations implicitly. Because it is not a matter of later AoO, “the earlier the better” approach that drives language learning curricula in many countries is not the answer unless students can interact in the target language for extensive amounts of time. Language practitioners and coordinators could incorporate curricular changes to replace “learning-about-language” time with “using-language” time. Considering studies reporting language use effects on L2 learners, adopting a language teaching methodology that focuses on communication, encouraging learners to live abroad, and administering tests that assess language use rather than proficiency could help. For example, Beatty-Martínez et al. (2020) observed that L2 learners of the same proficiency demonstrated differences in their sensitivity to code-switching rules (those code-switching more often were more sensitive to code-switching rules). This study suggests that L2 learners are able to take advantage of extensive L2 use.

Altogether, our results demonstrated that language proficiency and use are different constructs that have distinct consequences on bilingual language processing. This proposal is consistent with recent neurocognitive evidence showing differences between proficiency and use. For instance, Del Maschio et al. (2020) found that white matter microstructure increased with greater language use rather than AoO or proficiency. Similarly, other scholars reported that *later* AoO, a possible sign of greater language use, increased white matter microstructure (Nichols and Joanisse, 2016; DeLuca et al., 2019). Language use has also been associated with subcortical brain structures related to language management processes (DeLuca et al., 2019). Furthermore, proficiency and use seem to influence distinct brain areas: language use modulates areas linked to cognitive control and general comprehension and production, whereas language proficiency affects areas related to word learning and language selection (Fedeli et al., 2021). Turning our attention to the increased importance of language use over language proficiency in HSs, the few studies investigating the effects of language proficiency and use on HSs’ processing are consistent with our findings. Sagarra and Rodríguez (2022) reported no proficiency effects on HSs’ (or L2 learners’) morphosyntactic predictions, and Wartenburger et al. (2003) found no proficiency effects on HSs’ grammatical judgments. Although Hervais-Adelman et al. (2018) equated higher proficiency with greater volume of brain areas associated with language control in HSs, their results were based on a sui generis variable mixing proficiency and AoO, and brain measures of gray matter “volume” involving voxel-based morphometry are difficult to interpret. Instead of looking at gray matter volume, scholars can examine cortical thickness (more experience-related) and surface area (more innate) independently. Concerning the different role of language proficiency and use on HSs’ grammatical processing, Sagarra et al. (2021) observed that, while proficiency and use increased morphosyntactic predictions, language proficiency yielded faster predictions and more attention to gender suffixes (e.g., knowing that *−a* denotes feminine gender), and language use produced earlier predictions and more attention to inherent gender information in nouns lacking transparent gender suffixes (e.g., knowing that *pared* “wall” is feminine in Spanish). Finally, regarding language use affecting language processing in HSs but not L2 learners, Sagarra and Casillas (in progress) collected phonotactic frequency data with advanced HSs and L2 learners completing the same eye-tracking task with the same stimuli as the present study. They found that higher phonotactic frequency increased fixations on targets in HSs, but not in L2 learners. In light of the essential role that language use played on HS processing along with the distinct consequences of language proficiency and use on L2 and HS processing and prediction, future HS and L2 studies and placement tests should incorporate measures of language proficiency and language use.

## 7. Conclusion and theoretical implications

This study examined the role of AoO, language proficiency, and language use on stress-tense suffix associations involving a stressed syllable cuing a present suffix and an unstressed syllable cuing a preterit suffix in Spanish regular verbs by adult Spanish-English HSs, English-Spanish L2 learners, and Spanish monolinguals. Participants saw a paroxytone verb (*salta* “s/he jumps”) and an oxytone verb (*saltó* “s/he jumped”) side by side, heard a sentence containing one of the verbs, and selected the verb they had heard. In English disyllabic verbs, oxytones are more common, whereas in Spanish words, paroxytones are more typical. The two bilingual groups were uniform in their Spanish proficiency and use. Eye-tracking data indicated that all groups fixated on target verbs above chance before hearing the second syllable that contained the suffix, except the HSs in oxytones. Monolinguals fixated on targets more and earlier, but at a slower rate than HSs and L2 learners. In turn, HSs fixated on targets more and earlier than L2 learners, except in oxytones where HSs fixated on targets less and later than L2 learners. This was due to HSs’ high number of lexical competitors due to their double L1 lexica, rather than lexical frequency or AoO. Language proficiency accounted for prediction in HSs and L2 learners and interacted with language exposure: higher proficiency increased predictions of oxytones in HSs (HSs’ unpreferred condition) but of paroxytones in L2 learners (L2 learners’ unfavored condition). In contrast, language use only accounted for prediction in HSs: greater use increased their predictions of oxytones. We conclude that HSs’ lexical access depends more on the number of lexical competitors (co-activation of two L1 lexica) and type (phonotactic) frequency than on token (lexical) frequency or AoO. Finally, language use accounted for HS predictions more than proficiency.

Our findings inform theoretical models in phonology, lexical access, language processing, language prediction, and neurocognition. First, our data align with phonology models positing that adult L2 learners can acquire suprasegmental information different from their L1 (e.g., Van Leussen and Escudero, 2015; Flege and Bohn, 2021), and lexical access models determining that prosody influences how we activate and store words in our brain (e.g., Roll, 2015). Our results are also consistent with L2 processing models arguing that higher proficiency facilitates L2 morphological activation and allows learners to move from decompositional to full-storage lexical access (e.g., Bybee, 1985; Gonnerman et al., 2007). Moreover, our analyses indicate that HS lexical access depends on co-activation cognitive demands resulted from activating a large number of lexical competitors in their two L1 lexica. Our data do not provide evidence that HS’ unique processing patterns are due to reduced exposure to input (Montrul, 2008; Pires and Rothman, 2009; Polinsky, 2011) or to reduced current activation of their heritage language (Hulsen, 2000; Putnam and Sánchez, 2013). Our findings also fall in line with L1 (Kuperberg and Jaeger, 2016) and L2 (Kaan and Grüter, 2021) models claiming that prediction variability is partially caused by individual differences in “utility” and expand these models to HS populations. Utility refers to adopting a fight-or-flee approach to prediction. That is, listeners weigh the benefits (e.g., faster processing) of engaging in prediction against its cost (e.g., risking it to make incorrect predictions); if it is worth the risk, they predict; otherwise, they do not. Lastly, our conclusions are consonant with usage-based cognitive models advocating that native early and late bilingual listeners process and predict language probabilistically based on their individual language experiences, and that language proficiency and use are separate constructs that exert distinct effects on brain adaptations (DeLuca et al., 2020). To shed light on the causes of variability of bilingual language processing, future studies should include early and late bilinguals, online auditory implicit tasks, continuous (rather than categorical) measures of AoO, proficiency, use and exposure, and type and token frequency assessments. With a goal of increasing L2 learning in mind, language practitioners can provide learners with numerous opportunities to interact in the target language and L2 learners can live abroad to maximize actively using the L2. To conclude, the underpinning of bilingual language processing variability is built upon a simple yet tremendously fluid and powerful tenet: use it or lose it.

## Data availability statement

The original contributions presented in the study are included in the article/Supplementary material, and the following link: https://osf.io/2p46d/. Further inquiries can be directed to the corresponding author.

## Ethics statement

This study was reviewed and approved by Institutional Review Board at Rutgers, The State University of New Jersey, New Brunswick. The participants provided their written informed consent to participate in this study.

## Author contributions

NS conceived and designed the study, collected the data, wrote the Introduction, Background, Methods, Discussion, and Conclusion sections of the article. JC analyzed the data, wrote the Lexical stress, and Results sections of the article, and created the tables and figures. All authors contributed to the article and approved the submitted version.

## Conflict of interest

The authors declare that the research was conducted in the absence of any commercial or financial relationships that could be construed as a potential conflict of interest.

## Publisher’s note

All claims expressed in this article are solely those of the authors and do not necessarily represent those of their affiliated organizations, or those of the publisher, the editors and the reviewers. Any product that may be evaluated in this article, or claim that may be made by its manufacturer, is not guaranteed or endorsed by the publisher.

## Footnotes

^1^In both cases, the response variable, *use* or *proficiency* score, was a proportion. Thus, we used the beta distribution for the model likelihood with a logit linking function. The models included regularizing, weakly informative priors. See the Supplementary material for full details.

^2^GAMMs represent an extension to the linear model framework that allow non-linear functions called factor smooths to be applied to predictors. In this sense, the predictors can be classified into two types: parametric terms (equivalent to fixed effects in hierarchical model terminology) and smooth terms. Random smooths are conceptually similar to random slopes and intercepts in the mixed-effects regression framework (Winter and Wieling, 2016). Thus, they allow the by-subject trajectory shapes to vary as a function of a parametric effect and are essential in avoiding anti-conservative models.

^3^See Supplementary material for detailed information.

^4^More information regarding zero-inflated beta regression is available in the Supplementary material.
