# Supplementary material for: Practice beats age: co-activation shapes heritage speakers’ lexical access more than age of onset
Source: Front Psychol. 2023 Jun 12;14:1141174. doi: 10.3389/fpsyg.2023.1141174 (PMC10292756; doi:10.3389/fpsyg.2023.1141174)
Supplement: Supplementary file 1 [file Data_Sheet_1.pdf]

Supplementary Materials

1 Bayesian data analysis

This study employs Bayesian Data Analysis for quantitative inferential statistics. Specifically, this implies that we use Bayesian *credible intervals*—and other metrics—to draw statistical inferences. A Bayesian model calculates a posterior distribution, i.e., a distribution of plausible parameter values, given the data, a data-generating model, and any prior assumptions we have about those parameter values. Posterior distributions are computationally costly. For this reason, we use the Hamiltonian Markov Chain Monte Carlo algorithm to obtain a sample that includes thousands of values from the posterior distribution. In practical terms, what this means is that we do not calculate a single point estimate for an effect β, but rather we draw a sample of 4,000 plausible values for β. This allows us to quantify our uncertainty regarding β by summarizing the distribution of those values. We will use 4 statistics to describe the posterior distribution: (1) the posterior mean, (2) the highest density credible interval (HDI), (3) the proportion of the HDI that falls within a Region of Practical Equivalence (ROPE), and (4) the Maximum Probability of Effect (MPE). The posterior mean provides a point estimate for the distribution. The 95% highest density credible interval provides bounds for the effect. The ROPE designates a region of practical equivalence for a negligible effect and calculates the proportion of the HDI that falls within this interval.[5] The MPE calculates the proportion of the posterior distribution that is of the median's sign (or the probability that the effect is positive or negative). If, for instance, a hypothesis states that $\beta > 0$, we judge there to be *compelling evidence* for this hypothesis if the mean point estimate is a positive number, if the 95% credible interval of β does not contain 0 and is outside the ROPE by a reasonably clear margin, and the posterior $P(\beta > 0)$ is close to one. Together these four statistics allow us to quantify our uncertainty and provide an intuitive interpretation of any given effect. Consider a case in which the posterior mean of β is 100 and the 95% credible interval is [40, 160]. The interval tells us that we can be 95% certain the *true* value of β is between 40 and 160, given the data, our model, and our prior information. Furthermore, the interval allows us to specify areas of uncertainty. In this example, we can conclude that the effect is almost certain to be positive. The lower interval value of 40 tells us that 95% of the plausible values are greater than 40. We also note that the interval covers a wide range of values, thus we also conclude that we are not very certain about the size of the effect. This type of interpretation is not possible under a frequentist paradigm.

2 GAMMs

Supplementary Table 1. Group GAMM summary.

Effect	Term	Estimate	HDI	Rhat	Bulk ESS	Tail ESS
Population	Intercept	0.60	[0.44, 0.76]	1.00	797.53	1436.65
	HS	−0.30	[−0.53, −0.11]	1.01	744.97	1223.82
	L2	−0.43	[−0.64, −0.23]	1.00	708.42	761.10
	Oxytone	0.06	[0.05, 0.08]	1.00	3429.99	3735.29
	HS:Oxytone	−0.44	[−0.46, −0.42]	1.00	3469.26	3630.77
	L2:Oxytone	0.00	[−0.02, 0.02]	1.00	3114.31	3926.50
	Time	−0.26	[−0.26, −0.26]	1.00	3581.16	3934.31
	Time:Oxytone	−0.01	[−0.01, −0.01]	1.00	3865.39	3348.79
Grouping	sd(Intercept)	0.45	[0.40, 0.51]	1.00	1088.45	1923.43
Smooths	sds(Time)	0.03	[0.02, 0.06]	1.00	1620.98	2149.99
	sds(Time:Oxytone)	0.02	[0.01, 0.04]	1.00	2522.74	3383.84

Supplementary Table 2. Group prediction summary.

Group	Stress	Estimate	HDI	ROPE	PD
Monolingual	Paroxytone	0.64	[0.61, 0.68]	0.00	1.00
	Oxytone	0.66	[0.63, 0.70]	0.00	1.00
HS	Paroxytone	0.57	[0.54, 0.60]	0.00	1.00
	Oxytone	0.48	[0.45, 0.52]	0.97	0.85
L2	Paroxytone	0.54	[0.51, 0.57]	0.01	1.00
	Oxytone	0.56	[0.53, 0.59]	0.00	1.00

Supplementary Figure 1. Marginal slope estimates 200ms after the offset of 1st syllable for monolinguals, heritage speakers, and L2 learners in paroxytone (CANto) and oxytone (canTO) conditions. Group marginal slopes pairwise comparisons summary.

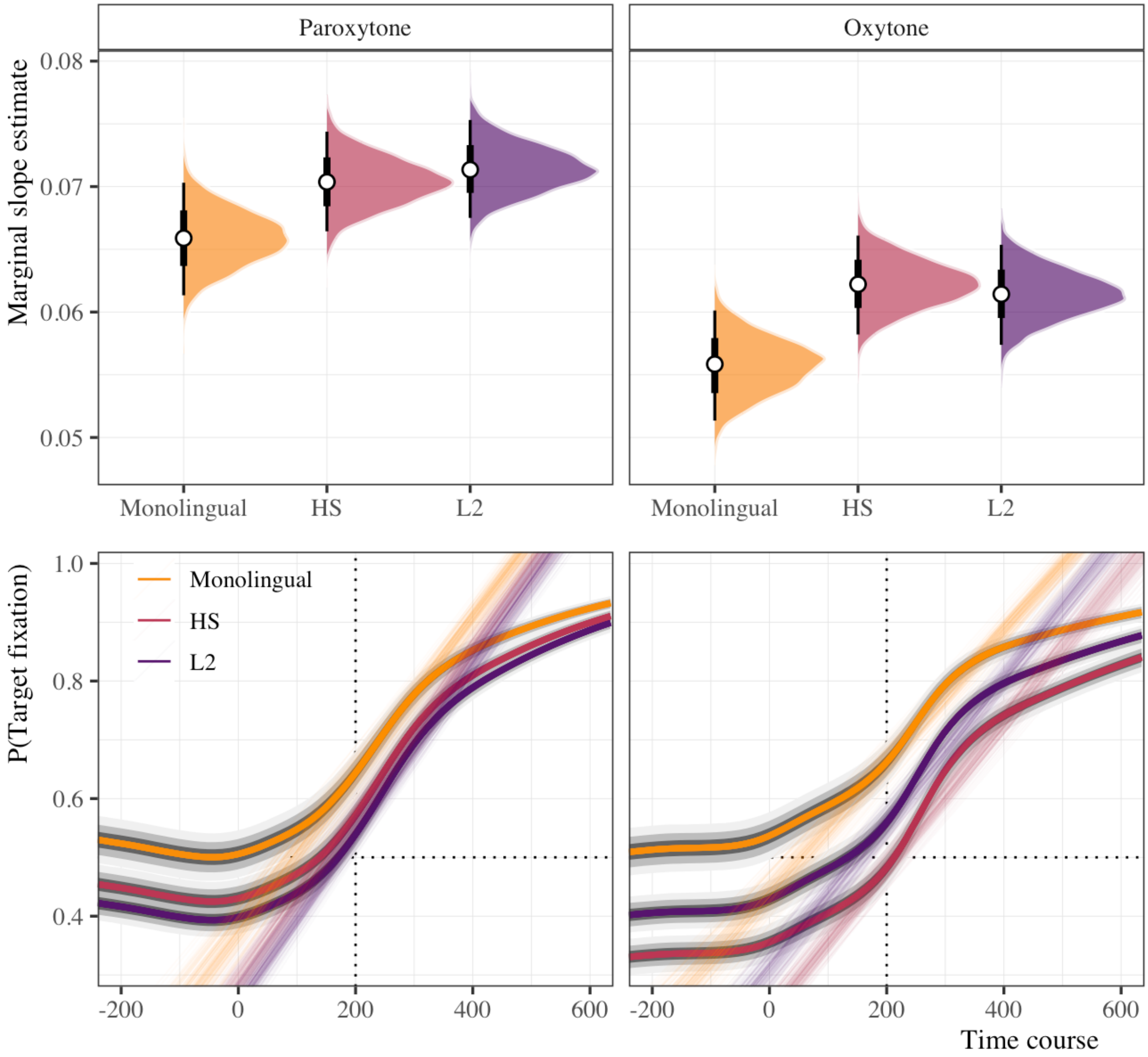

Supplementary Table 3. Marginal slopes pairwise comparisons.

Stress	Comparison	Estimate	HDI	ROPE	PD
Paroxytone	HS - L2	-0.001	[-0.003, 0.000]	0.514	0.922
	Mono – HS	-0.004	[-0.008, -0.001]	0.000	0.999
	Mono - L2	-0.006	[-0.009, -0.003]	0.000	1.000
Oxytone	HS - L2	0.001	[0.000, 0.002]	0.697	0.957
	Mono - HS	-0.006	[-0.010, -0.004]	0.000	1.000
	Mono - L2	-0.006	[-0.009, -0.003]	0.000	1.000

Supplementary Figure 2. Probability of fixating on target as a function of stress for the bilingual groups. The time course is centered around the target syllable offset (point 0). Vertical lines represent 200ms after target syllable offset. Lines represent paroxytone ('CANta', green) and oxytone ('canTO', orange) conditions plus 95%, 80%, and 50% credible intervals. Pairwise difference smooths for paroxytone and oxytone items. From dark to light, colors represent 95%, 80%, 70%, 60%, 50%, 35% and 10% credible intervals.

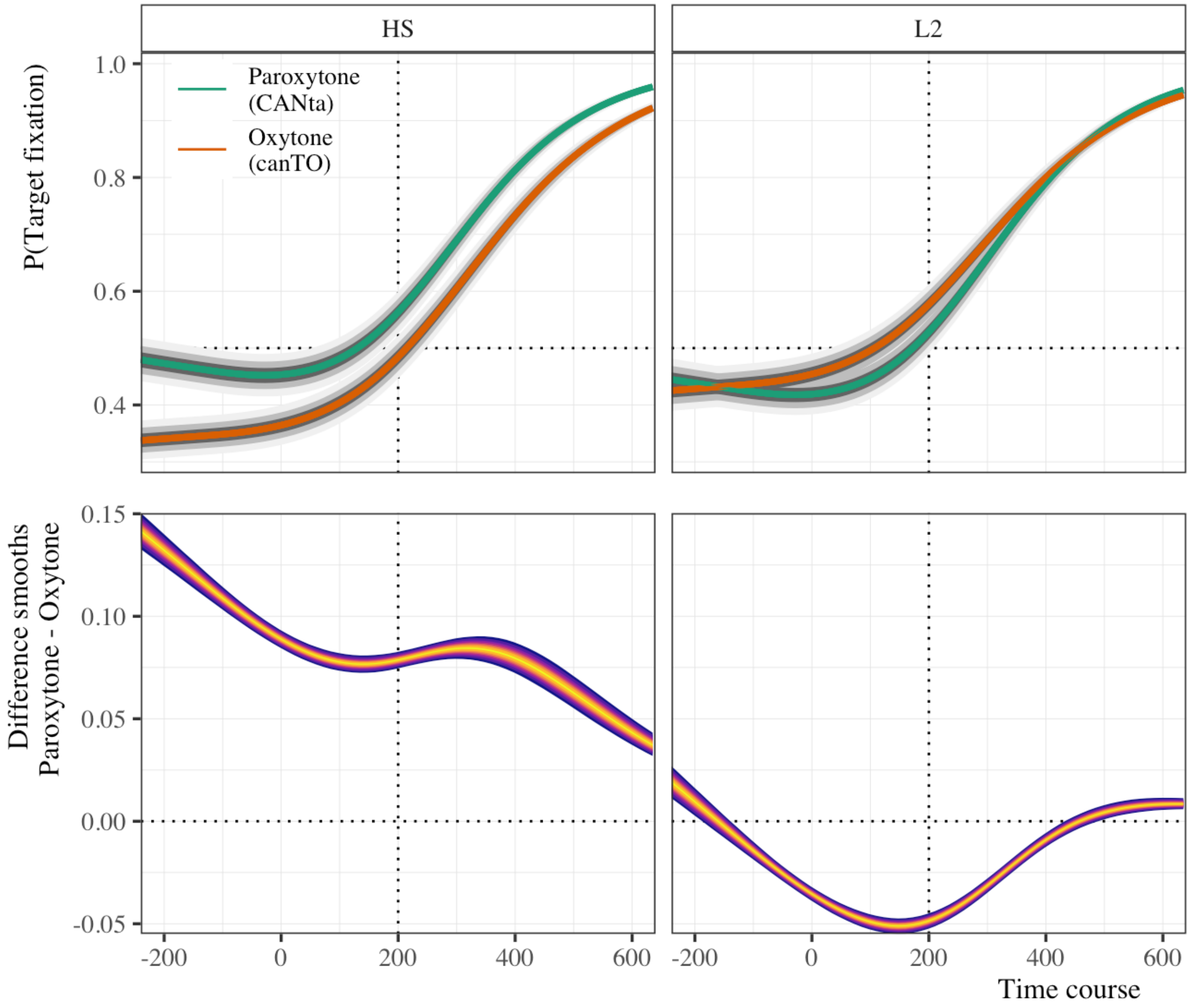

Supplementary Table 4. Bilingual GAMM summary.

Effect	Term	Estimate	HDI	Rhat	Bulk ESS	Tail ESS
Population	Intercept	0.39	[0.24, 0.54]	1	922.53	1521.88
	L2	−0.14	[−0.35, 0.07]	1	987.96	1527.22
	Oxytone	−0.39	[−0.41, −0.38]	1	3526.63	3831.69
	Proficiency	0.03	[−0.08, 0.13]	1	1295.05	2034.59
	Use	0.01	[−0.10, 0.11]	1	1325.31	1976.81
	L2:Oxytone	0.51	[0.50, 0.53]	1	3826.82	3752.23
	Proficiency:Use	−0.09	[−0.21, 0.02]	1	1334.78	2066.52
	Time	1.49	[1.48, 1.51]	1	3957.52	3774.16
	Time:Oxytone	0.00	[−0.02, 0.02]	1	3741.31	3649.01
Grouping	sd(Intercept)	0.49	[0.42, 0.57]	1	1433.13	2323.04
Smooths	sds(Time)	1.42	[0.37, 3.74]	1	3091.95	3338.58
	sds(Time:Oxytone)	0.91	[0.10, 3.29]	1	2501.40	2420.84

Supplementary Figure 3. Forest plot of the bilingual GAMM. The horizontal axis represents the models estimates in log-odds. The vertical axis lists the terms estimated in the model. The points illustrate the posterior mean along with the 66% and 95% HDI. The vertical faceting separates the estimates into parametric and non-parametric population-level effects, group-level effects, and smooth terms.

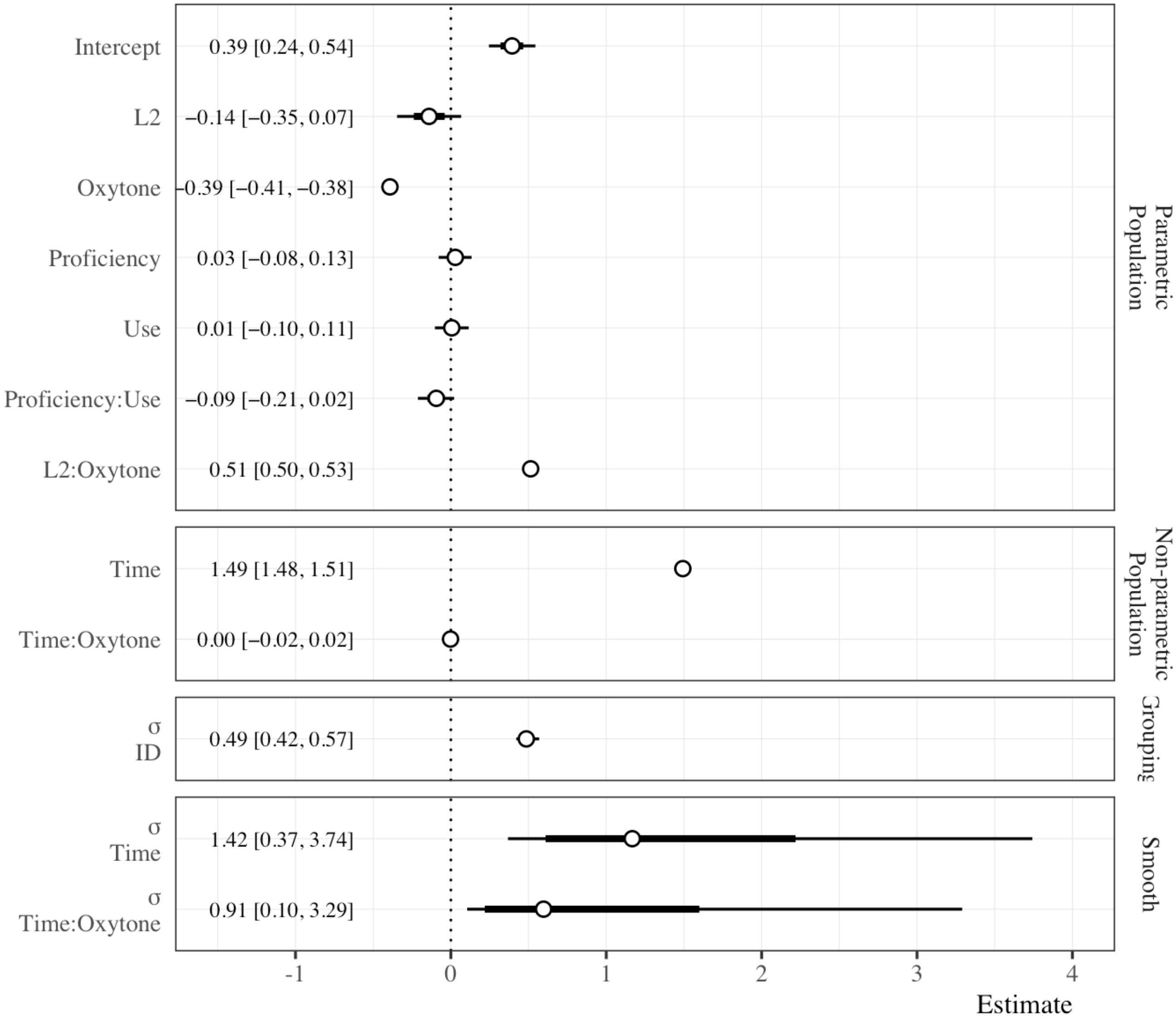

3 Proficiency and use

3.1. General descriptives.

Figure 4 illustrates the marginal mean differences (L2 - HS) for each metric. The plot provides visual evidence supporting the notion that the two groups are similar in their proficiency in and use of Spanish. For both metrics, the entirety of the 95% HDI falls within the ROPE (±0.1). Figure 11 complements Table 1 in the manuscript.

Supplementary Figure 4. Marginal mean estimates plot.

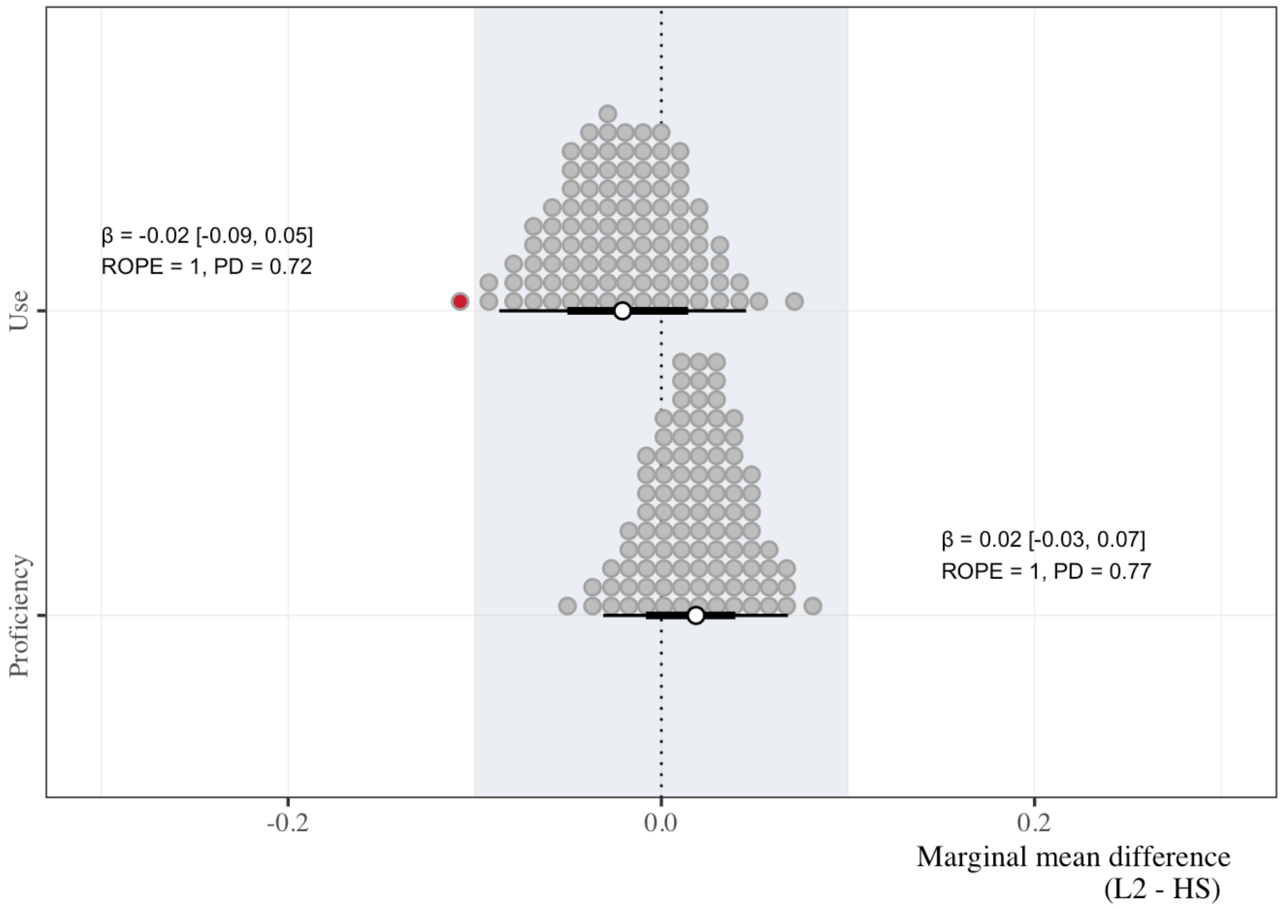

3.2. Zero-inflated beta regression.

Table 5 provides a model summary of the zero-inflated beta regression conducted in proficiency and use. This table provides the same information provided in the forest plot.

Supplementary Table 5. Model output from zero-inflated beta regression.

Effect	Term	Estimate	HDI	ROPE	PD
Population-level	Intercept	0.11	[0.01, 0.22]	0.39	0.99
	phi Intercept	2.12	[1.81, 2.47]	0.00	1.00
	zi Intercept	−4.80	[−7.33, −3.36]	0.00	1.00
	Group	−0.05	[−0.16, 0.05]	0.83	0.83
	Stress	−0.02	[−0.12, 0.08]	0.97	0.63
	Proficiency	0.07	[−0.05, 0.19]	0.69	0.87
	Use	0.02	[−0.09, 0.12]	0.96	0.63
	Group:Stress	0.17	[0.06, 0.27]	0.08	1.00
	Group:Use	−0.13	[−0.23, −0.02]	0.30	0.99
	Group:Proficiency	−0.03	[−0.15, 0.10]	0.91	0.66
	Group:Use:Proficiency	−0.03	[−0.15, 0.09]	0.88	0.67
Group-level	sd(Intercept)	0.13	[0.01, 0.32]	0.42	1.00
	sd(Stress)	0.12	[0.01, 0.31]	0.45	1.00
	sd(phi Intercept)	0.64	[0.22, 1.05]	0.00	1.00
	sd(phi Stress)	0.33	[0.02, 0.74]	0.13	1.00
	sd(zi Intercept)	0.80	[0.03, 2.46]	0.06	1.00
	sd(zi Stress)	0.83	[0.03, 2.55]	0.05	1.00
	cor(Intercept, Stress)	−0.19	[−0.97, 0.91]	0.09	0.65
	cor(phi Intercept, phi Stress)	0.41	[−0.63, 0.97]	0.09	0.82
	cor(zi Intercept, zi Stress)	0.11	[−0.90, 0.95]	0.12	0.59

Figure 5 shows the marginal mean estimates of fixating on a target when marginalizing over proficiency and use. This plot resembles Figure 4, however, it differs in that it depicts estimates holding proficiency and use constant at their respective averages.

Supplementary Figure 5. Marginal mean estimates of target fixations for HS and L2 groups as a function of stress. Language proficiency and use are held constant at z = 0.

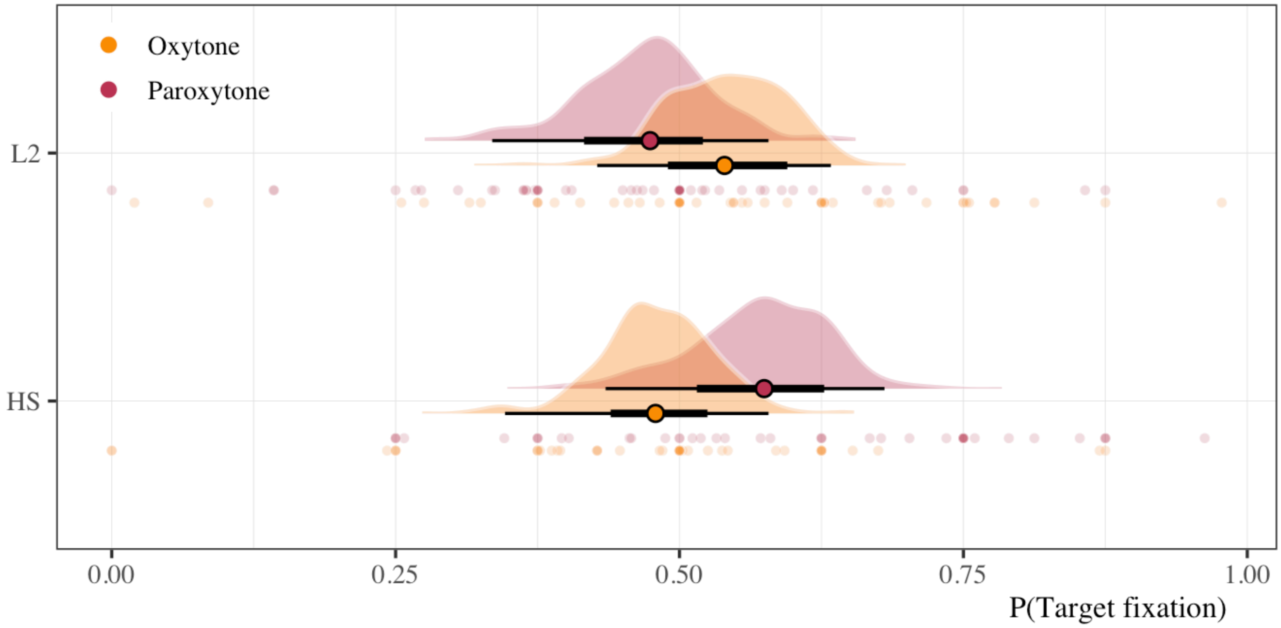

Figure 6 plots target fixation as a function of group, use, and proficiency in the paroxytone and oxytone stress conditions, as well as marginalizing over stress. This figure complements the zero-inflated beta regression analysis provided in the section on proficiency and use.

Supplementary Figure 6. Use and proficiency in Spanish.

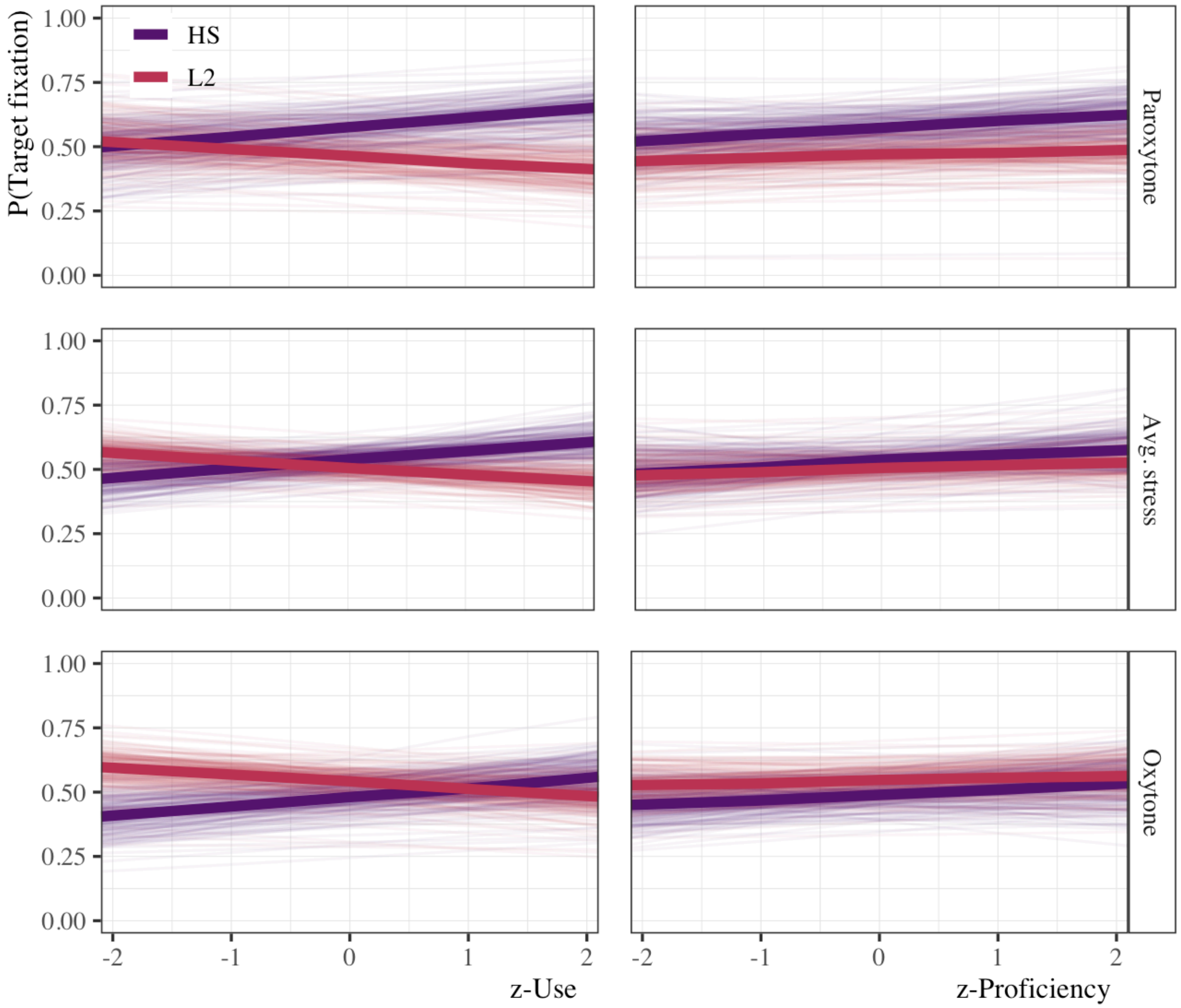

4 Reproducibility information

About this document

This document was written in `quarto`.

Session info

```
 setting  value
 version  R version 4.2.1 (2022-06-23)
 os       macOS Big Sur ... 10.16
 system   x86_64, darwin17.0
 ui       X11
 language (EN)
 collate  en_US.UTF-8
 ctype    en_US.UTF-8
 tz       America/New_York
 date     2022-10-11
 pandoc   2.18 @ /Applications/RStudio.app/Contents/MacOS/quarto/bin/tools/ (via
rmarkdown)
               loadedversion       date
abind                  1.4-5 2016-07-21
arrayhelpers           1.1-0 2020-02-04
assertthat             0.2.1 2019-03-21
backports              1.4.1 2021-12-13
base64enc              0.1-3 2015-07-28
bayesplot              1.9.0 2022-03-10
bayestestR            0.12.1 2022-05-02
bit                    4.0.4 2020-08-04
bit64                  4.0.5 2020-08-30
bridgesampling         1.1-2 2021-04-16
brms                  2.17.0 2022-04-13
Brobdingnag            1.2-7 2022-02-03
cachem                 1.0.6 2021-08-19
callr                  3.7.2 2022-08-22
cellranger             1.1.0 2016-07-27
checkmate              2.1.0 2022-04-21
cli                    3.3.0 2022-04-25
coda                  0.19-4 2020-09-30
codetools             0.2-18 2020-11-04
colorspace             2.0-3 2022-02-21
colourpicker           1.1.1 2021-10-04
crayon                 1.5.1 2022-03-26
crosstalk              1.2.0 2021-11-04
datawizard             0.5.1 2022-08-17
DBI                    1.1.3 2022-06-18
devtools               2.4.4 2022-07-20
digest                0.6.29 2021-12-01
distributional         0.3.0 2022-01-05
dplyr                  1.0.9 2022-04-28
DT                      0.24 2022-08-09
dygraphs             1.1.1.6 2018-07-11
ellipsis               0.3.2 2021-04-29
emmeans              1.8.1-1 2022-09-08
estimability           1.4.1 2022-08-05
evaluate                0.16 2022-08-09
fansi                  1.0.3 2022-03-24
farver                 2.1.1 2022-07-06
```

```
fastmap              1.1.0 2021-01-25
forcats              0.5.2 2022-08-19
fs                   1.5.2 2021-12-08
generics             0.1.3 2022-07-05
ggdist               3.2.0 2022-07-19
ggplot2              3.3.6 2022-05-03
ggridges             0.5.3 2021-01-08
glue                 1.6.2 2022-02-24
gridExtra              2.3 2017-09-09
gtable               0.3.0 2019-03-25
gtools               3.9.3 2022-07-11
here                 1.0.1 2020-12-13
highr                  0.9 2021-04-16
hms                  1.1.2 2022-08-19
htmltools            0.5.3 2022-07-18
htmlwidgets          1.5.4 2021-09-08
httpuv               1.6.5 2022-01-05
igraph               1.3.4 2022-07-19
inline              0.3.19 2021-05-31
insight             0.18.2 2022-08-10
janitor              2.1.0 2021-01-05
jsonlite             1.8.0 2022-02-22
knitr                 1.40 2022-08-24
later                1.3.0 2021-08-18
lattice            0.20-45 2021-09-22
lifecycle            1.0.1 2021-09-24
loo                  2.5.1 2022-03-24
lubridate            1.8.0 2021-10-07
magrittr             2.0.3 2022-03-30
markdown               1.1 2019-08-07
Matrix               1.5-0 2022-09-10
matrixStats         0.62.0 2022-04-19
memoise              2.0.1 2021-11-26
mime                  0.12 2021-09-28
miniUI             0.1.1.1 2018-05-18
munsell              0.5.0 2018-06-12
mvtnorm              1.1-3 2021-10-08
nlme               3.1-157 2022-03-25
patchwork            1.1.2 2022-08-19
pillar               1.8.1 2022-08-19
pkgbuild             1.3.1 2021-12-20
pkgconfig            2.0.3 2019-09-22
pkgload              1.3.0 2022-06-27
plyr                 1.8.7 2022-03-24
posterior            1.3.0 2022-08-15
prettyunits          1.1.1 2020-01-24
processx             3.7.0 2022-07-07
profvis              0.3.7 2020-11-02
promises           1.2.0.1 2021-02-11
ps                   1.7.1 2022-06-18
purrr                0.3.4 2020-04-17
R6                   2.5.1 2021-08-19
Rcpp                 1.0.9 2022-07-08
RcppParallel         5.1.5 2022-01-05
readr                2.1.2 2022-01-30
readxl               1.4.1 2022-08-17
remotes              2.4.2 2021-11-30
reshape2             1.4.4 2020-04-09
```

```
rlang                  1.0.4 2022-07-12
rmarkdown               2.16 2022-08-24
rprojroot              2.0.3 2022-04-02
rstan                 2.21.5 2022-04-11
rstantools             2.2.0 2022-04-08
rstudioapi              0.14 2022-08-22
scales                 1.2.1 2022-08-20
sessioninfo            1.2.2 2021-12-06
shiny                  1.7.2 2022-07-19
shinyjs                2.1.0 2021-12-23
shinystan              2.6.0 2022-03-03
shinythemes            1.2.0 2021-01-25
snakecase             0.11.0 2019-05-25
StanHeaders         2.21.0-7 2020-12-17
stringi                1.7.8 2022-07-11
stringr                1.4.1 2022-08-20
svUnit                 1.0.6 2021-04-19
tensorA               0.36.2 2020-11-19
threejs                0.3.3 2020-01-21
tibble                 3.1.8 2022-07-22
tidybayes              3.0.2 2022-01-05
tidyr                  1.2.0 2022-02-01
tidyselect             1.1.2 2022-02-21
tzdb                   0.3.0 2022-03-28
urlchecker             1.0.1 2021-11-30
usethis                2.1.6 2022-05-25
utf8                   1.2.2 2021-07-24
vctrs                  0.4.1 2022-04-13
vroom                  1.5.7 2021-11-30
withr                  2.5.0 2022-03-03
xfun                    0.32 2022-08-10
xtable                 1.8-4 2019-04-21
xts                   0.12.1 2020-09-09
yaml                   2.3.5 2022-02-21
zoo                   1.8-10 2022-04-15
```

5 Data repository

https://osf.io/jqzwy/
